# Cyclic Peptidic Furin Inhibitors Developed by Combinatorial Chemistry

**DOI:** 10.1021/acsmedchemlett.3c00008

**Published:** 2023-03-15

**Authors:** Agata Gitlin-Domagalska, Dawid Dębowski, Aleksandra Maciejewska, Sergey Samsonov, Martyna Maszota-Zieleniak, Natalia Ptaszyńska, Anna Łęgowska, Krzysztof Rolka

**Affiliations:** † Department of Molecular Biochemistry, Faculty of Chemistry, University of Gdańsk, 80-308 Gdańsk, Poland; ‡ Department of Theoretical Chemistry, Faculty of Chemistry, University of Gdańsk, 80-308 Gdańsk, Poland

**Keywords:** furin, sunflower trypsin inhibitor-1, inhibitor, peptide library, combinatorial chemistry

## Abstract

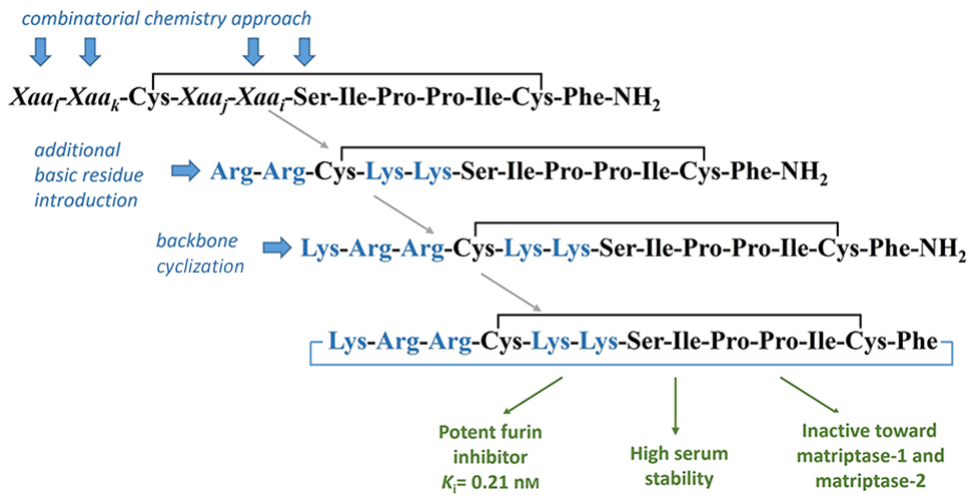

Furin is a human serine protease responsible for activating numerous physiologically relevant cell substrates and is also involved in the development of various pathological conditions, including inflammatory diseases, cancers, and viral and bacterial infections. Therefore, compounds with the ability to inhibit furin’s proteolytic action are regarded as potential therapeutics. Here we took the combinatorial chemistry approach (library consisting of 2000 peptides) to obtain new, strong, and stable peptide furin inhibitors. The extensively studied trypsin inhibitor SFTI-1 was used as a leading structure. A selected monocylic inhibitor was further modified to finally yield five mono- or bicyclic furin inhibitors with values of *K*_i_ in the subnanomolar range. Inhibitor **5** was the most active (*K*_i_ = 0.21 nM) and significantly more proteolytically resistant than the reference furin inhibitor described in the literature. Moreover, it reduced furin-like activity in PANC-1 cell lysate. Detailed analysis of furin–inhibitor complexes using molecular dynamics simulations is also reported.

Furin (also known as paired basic amino acid cleaving enzyme (PACE)) is a type I transmembrane serine protease that belongs to the family of proprotein convertase subtilisin/kexin-type enzymes. Furin recognizes the consensus recognition motif in the primary structure of its protein substrates, Arg-Xaa-Lys/Arg-Arg↓ (RX_aa_K/RR↓, where the arrow indicates the cleavage site and X_aa_ corresponds to any amino acid), and hydrolyzes the peptide bond after Arg located at the substrate P1 position, which largely determines enzyme specificity.^1−3^ High levels of furin are found in the salivary glands, liver, and bone marrow.^4^ It is located mainly on the cell surface and in two subcellular localizations: early endosomes and the trans-Golgi network, where it cleaves and activates mostly host cell substrates, e.g., growth factors, neuropeptides, hormones, adhesion molecules, blood coagulation factors, and receptors.^5^ On the contrary, on the cell surface it activates cellular proteins involved in cell migration and tumor metastasis and cleaves external pathogenic substrates. Beside its physiological relevance, furin is also involved in the development of various inflammatory diseases, cancers, viral and bacterial infections, atherosclerosis, and neurodegenerative disorders.^3,6^ Regarding viruses, furin induces infection through processing of their surface glycoproteins (e.g., S-protein). Furin-mediated cleavage has been reported for glycoproteins produced by numerous evolutionarily diverse viruses, e.g., HIV, influenza, dengue, Ebola, or Marburg.^3,7^ Thus, furin is regarded as a potential drug target in various viral diseases.

Various covalent and non-covalent furin inhibitors, including engineered serpin (α1-PDX), peptidic chloromethyl ketones, 2,5-dideoxystreptamine derivatives, polyarginines, and peptidomimetics with a *C*-terminal decarboxylated 4-amidinobenzylamide (4-amba) group have been reported to date.^3,5,8,9^ Significantly, the therapeutic potential of most of them is seriously limited due to low cell permeability (e.g., large or highly charged molecules), proteolytic instability (peptides^10^), or insufficient specificity, resulting in cellular toxicity (irreversible peptidyl chloromethyl ketone and some reversible multibasic inhibitors containing 4-amba^5,11^). Due to proteolysis, opsonization, and agglutination, free peptides are not systemically stable without additional modifications.^12^

In this study, we obtained new cyclic, peptide-based, strong, stable furin inhibitors. Their structures were designed based on the sunflower trypsin inhibitor 1 (SFTI-1)^14^ analogue developed recently by Fittler et al.^13^ (Figure 1, named here the *Fittler inhibitor* (FI)). The relative stability, molecular weight (1513 Da) between those of small therapeutics (<500 Da) and biologics (>5000 Da), and the compact and rigid structure (two cyclic motifs, i.e., a disulfide bond and the continuous peptide backbone) make SFTI-1 an attractive framework for designing peptides with therapeutic potential.^15^ To identify preferred amino acids at the P1, P2, P4, and P5 positions, we took the combinatorial chemistry approach using monocyclic SFTI-1 as a leading structure. This combines a split and mix strategy of chemical synthesis of peptide libraries with their iterative deconvolution (screening) in solution. We have applied this particular deconvolution approach, developed in 1991 by Houghten et al.,^16^ to find substrates and inhibitors of various proteinases.^17−20^ To date, several reports have been published describing the identification of various furin inhibitors (e.g., polyarginines,^21−23^ multileucine peptides,^24^ and substrate-based inhibitors containing the H5N1 cleavage site^10^) through a combinatorial strategy. Significantly, they are based on a different approach than our deconvolution method used to identify individual compounds from synthetic libraries, known as positional scanning.^25^ After selecting the most active inhibitors, we extended their sequences by attaching a basic amino acid to their *N*-termini. To improve the stability of the obtained inhibitors, we synthesized their “head-to-tail” cyclic analogues. Finally, we applied molecular dynamics (MD) simulations to identify essential interactions of the selected inhibitors with the cognate enzyme.

**Figure 1:**
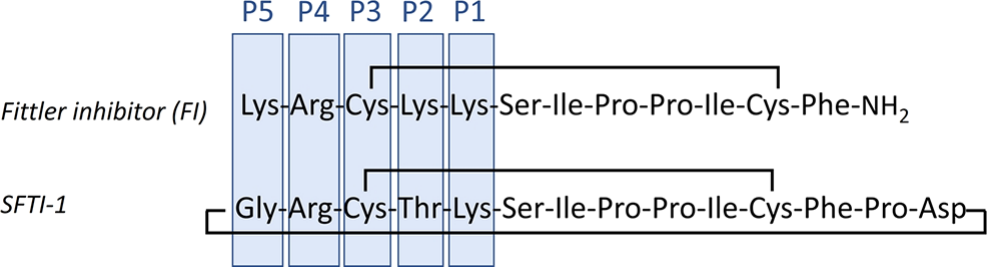
Structures of the Fittler inhibitor^13^ and SFTI-1.^14^

The general formula of the peptide amide library was *Xaa*_*l*_-*Xaa*_*k*_-Cys(&)-*Xaa*_*j*_-*Xaa*_*i*_-Ser-Ile-Pro-Pro-Ile-Cys(&)-Phe-NH_2_. In the substrate P1 position (Xaa_i_) two proteinogenic amino acids (Lys and Arg) were evaluated. The list of 10 amino acids, both proteinogenic and non-proteinogenic, introduced at positions P2 (Xaa_j_), P4 (Xaa_k_), and P5 (Xaa_l_) is provided in Figure 2.

**Figure 2:**
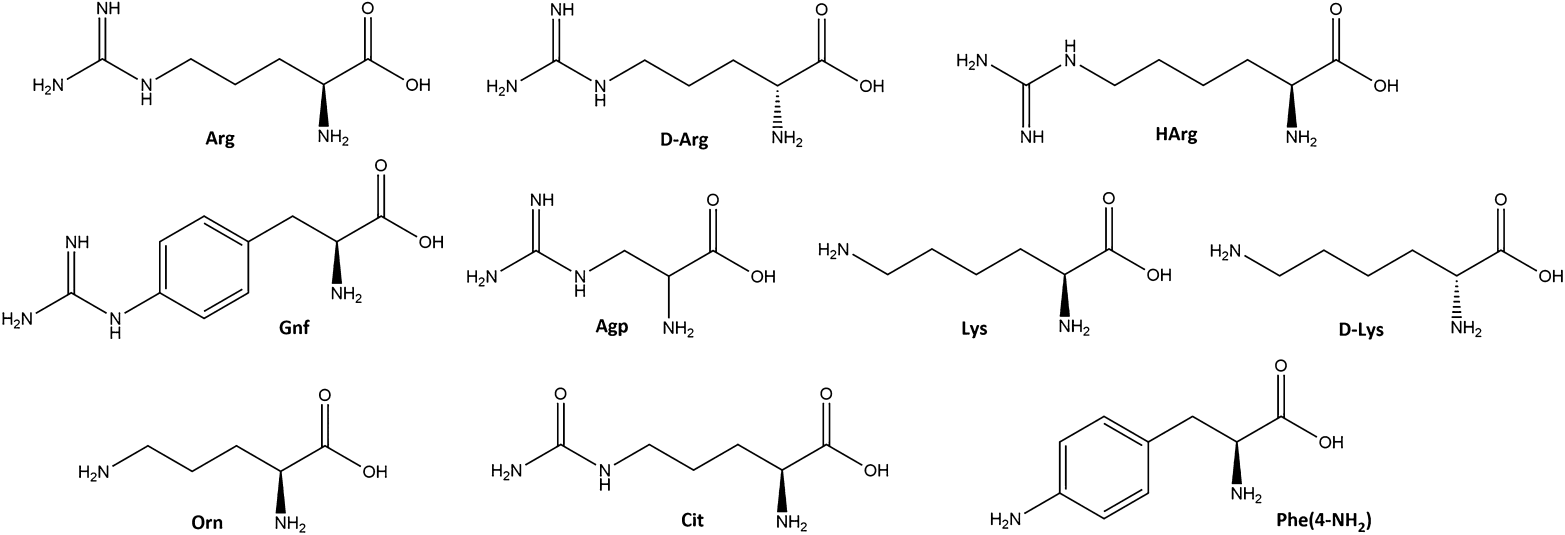
Amino acids used in the peptide library. hArg is homoarginine; Agp is 2-amino-3-guanidinopropionic acid; Gnf is 4-guanidinophenylalanine; Orn is ornithine; Cit is citrulline; Phe(4-NH_2_) is 4-aminophenylalanine.

Each of the sublibraries was screened for inhibitory activity against human recombinant furin in the presence of the fluorogenic substrate Pyr-Arg-Thr-Lys-Arg-AMC (where Pyr is pyroglutamic acid and AMC is 7-amino-4-methylcoumarin). Initially, each sublibrary was screened in a 2.2 ng/mL stock solution (Figure 3). If the obtained results did not clearly distinguish the most potent sublibrary, an additional analysis was conducted at lower concentrations.

**Figure 3:**
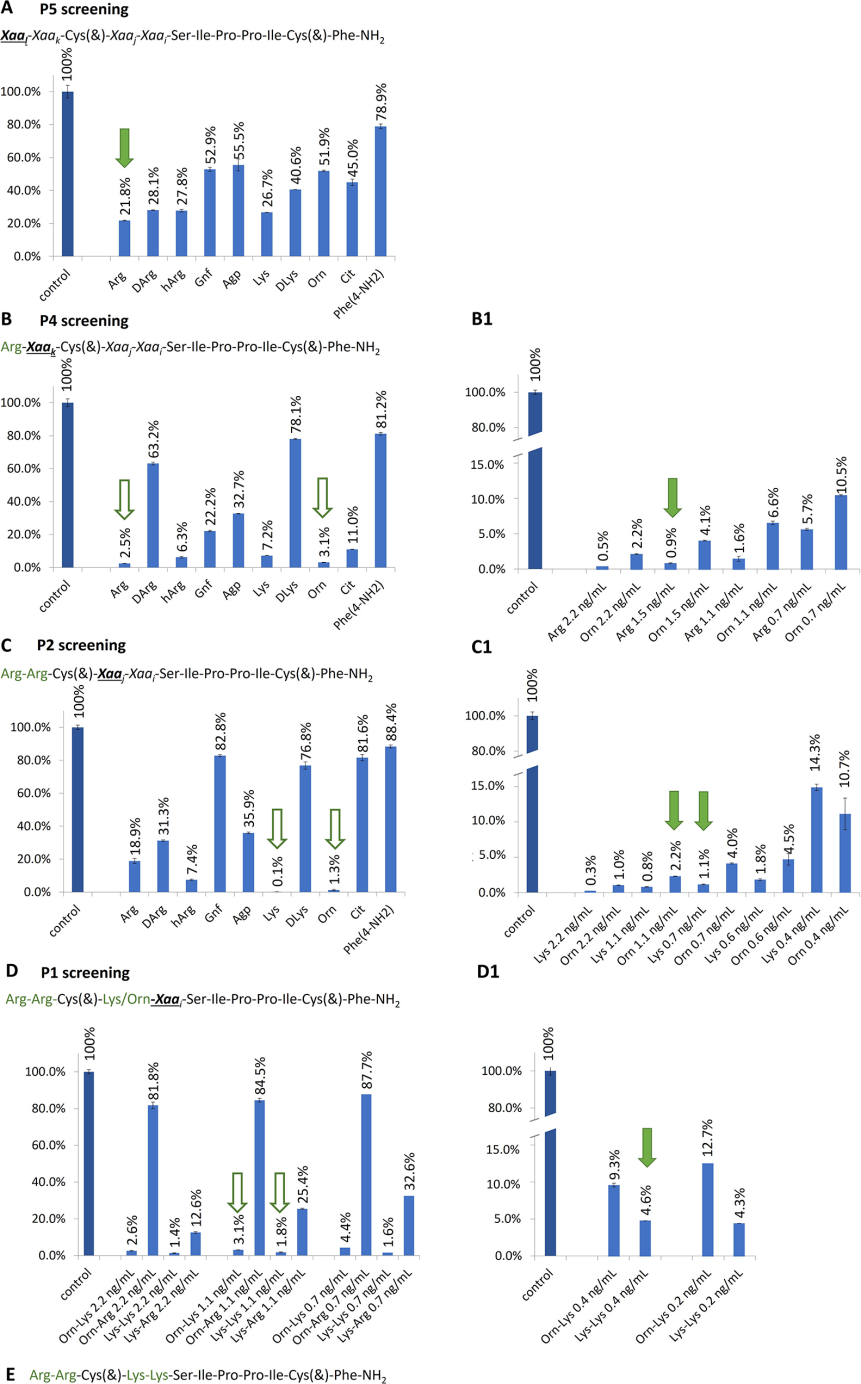
(A, B, C, D) Deconvolution of peptide libraries against furin. (B1, C1, D1) Additional assays with more concentrations are presented for P4, P2, and P1, respectively. Contour arrows indicate the most potent sublibraries that were subjected to additional enzymatic assay. Filled arrows point to amino acid residues selected for the next step of deconvolution. Xaa is any amino acid; hArg is homoarginine; Agp is 2-amino-3-guanidinopropionic acid; Gnf is 4-guanidinophenylalanine; Orn is ornithine; Cit is citrulline; Phe(4-NH_2_) is 4-aminophenylalanine. (E) Sequence of the most potent furin inhibitor selected upon library deconvolution.

The first library consisted of 10 sublibraries, each with a particular amino acid at the P5 position (Xaa_l_) (Figure 3), an equimolar mixture of those amino acids at positions Xaa_j_ and Xaa_k_, and a mixture of Lys and Arg at position Xaa_i_. No significant preference was revealed upon the first deconvolution step (Figure 3A). Sublibraries with Arg, d-Arg, hArg, and Lys presented comparable inhibitory potencies against furin. However, since the lowest enzyme activity (at least 5% lower than for other residues) was observed for the sublibrary with Arg at the *N*-terminus, this amino acid was fixed at the P5 position (Xaa_l_) in subsequent selection cycles. The next library, with Arg at P5, was composed of 10 sublibraries with particular amino acids at the P4 position (Xaa_k_). The strongest inhibitory activity was reported in the case of Arg and Orn (Figure 3B). More detailed analysis of these sublibraries revealed that Arg at P4 provides 2–4 times stronger furin inhibition than Orn (Figure 3B1). Therefore, Arg was selected and fixed in this position during further deconvolution.

Analysis of the P2 position revealed comparable preferences for Lys and its non-proteinogenic analogue Orn (Figure 3C). Though more detailed analysis (Figure 3C1) indicated that Lys was slightly more active, we decided to include both amino acids in the final deconvolution process. At the P1 position only two residues were tested (Arg and Lys) due to known furin substrate specificity.^3^ Four sublibraries with Arg fixed at P5 and P4 and either Lys or Orn at P2 were assessed. The greatest inhibition was observed for sublibraries with Lys at P1, while those with Arg were significantly less potent (Figure 3D). Comparison of two peptides with various amino acids at P2, Orn or Lys, revealed that the latter inhibitor, i.e., Arg-Arg-Cys(&)-Lys-Lys-Ser-Ile-Pro-Pro-Ile-Cys(&)-Phe-NH_2_ (inhibitor **1**), was 2–3 times more active (Figure 3D1). Thus, this peptide was selected for further optimization.

Next, we decided to extend the primary sequence of inhibitor **1** by attaching a Lys or Arg residue to its *N*-terminus (inhibitors **2** and **3**). Using X-ray crystallography, it was shown that a basic amino acid at the P6 position has a positive impact on the interaction with furin.^26^ Moreover, in many natural furin substrates and some synthetic inhibitors (polyarginines and polylysines),^22^ basic amino acids are overrepresented at the P5 and P6 positions. We also synthesized “head-to-tail” cyclic analogues of these peptides, i.e., **5** and **6**, as well as a bicyclic variant of the library-derived peptide **1**, i.e., peptide **4** (Table 1).

**Table 1:** Sequences of Synthesized Peptides^a^

peptide	sequence	monoisotopic mass [M + H]^+^	determined [M + H]^+^
**1**	Arg-Arg-Cys(&)-Lys-Lys-Ser-Ile-Pro-Pro-Ile-Cys(&)-Phe-NH_2_	1444.804	1444.781
**2**	Lys-Arg-Arg-Cys(&)-Lys-Lys-Ser-Ile-Pro-Pro-Ile-Cys(&)-Phe-NH_2_	1572.898	1572.965
**3**	Arg-Arg-Arg-Cys(&)-Lys-Lys-Ser-Ile-Pro-Pro-Ile-Cys(&)-Phe-NH_2_	1600.905	1600.970
**4**	&^1^Arg-Arg-Cys(&^2^)-Lys-Lys-Ser-Ile-Pro-Pro-Ile-Cys(&^2^)-Phe&^1^	1427.777	1427.766
**5**	&^1^Lys-Arg-Arg-Cys(&^2^)-Lys-Lys-Ser-Ile-Pro-Pro-Ile-Cys(&^2^)-Phe&^1^	1555.872	1555.901
**6**	&^1^Arg-Arg-Arg-Cys(&^2^)-Lys-Lys-Ser-Ile-Pro-Pro-Ile-Cys(&^2^)-Phe&^1^	1583.878	1583.875
FI	Lys-Arg-Cys(&)-Lys-Lys-Ser-Ile-Pro-Pro-Ile-Cys(&)-Phe-NH_2_	1416.773	1416.813

a The cyclization is indicated by (&) according to the recommendation of Spengler et al.^27^ FI is the Fittler inhibitor,^13^ acting as a reference peptide. Monocyclic peptides have a *C*-terminal amide group.

Initially, the activities of the furin inhibitors were screened at three concentrations, i.e., 2.4, 24.0, and 77.0 nM (Figure 4). For selected peptides **3**, **5**, and **6** and the reference FI, the inhibitory constants (*K*_i_) were calculated (Table 2). Except for the bicyclic peptide **4**, all of the inhibitors almost completely abolished the enzyme activity at the highest tested concentration. Peptide **4** was explicitly the weakest inhibitor in the series, also when compared with its monocyclic disulfide-bridged counterpart **1**. When the inhibitory activities were analyzed at the lowest tested concentration (2.4 nM), three peptides (**3**, **5**, and **6**) reduced the furin activity to less than 50% of its initial noninhibited activity. All of them have an additional basic amino acid at their *N*-termini (in MD simulations this is depicted as a residue with number “0”). Among them, two peptides share the same sequence, i.e., disulfide-bridged peptide **3** (*K*_i_ = 0.27 nM) and its bicyclic analogue peptide **6** (*K*_i_ = 0.25 nM). Such close inhibitory potency was not observed in the case of an analogous pair, i.e., **2** and **5**. Monocyclic **2** was less active than its bicyclic variant **5**. Significantly, the latter peptide was the strongest furin inhibitor (*K*_i_ = 0.21 nM). The reference FI displayed lower activity with *K*_i_ = 0.38 nM and, as shown in Figure 4, its inhibitory potency was comparable with that of peptide **1**. The two disulfide-bridged peptides, FI and **1**, differ only at the P4 position, having Lys and Arg, respectively. Furthermore, our compounds extend the relatively short list of known cyclic furin inhibitors, which includes the truncated analogue of SFTI-1 (FI), with a *K*_i_ value of 0.49 nM;^13^ cyclic polyarginines, with *K*_i_ values in the range of 0.1–1 μM;^28^ multi-Leu octapeptide, with a *K*_i_ close to 20 nM;^29^ and macrocyclic peptides with a *C*-terminal 4-amba group, with *K*_i_ in the nanomolar or even subnanomolar range.^30^

**Figure 4:**
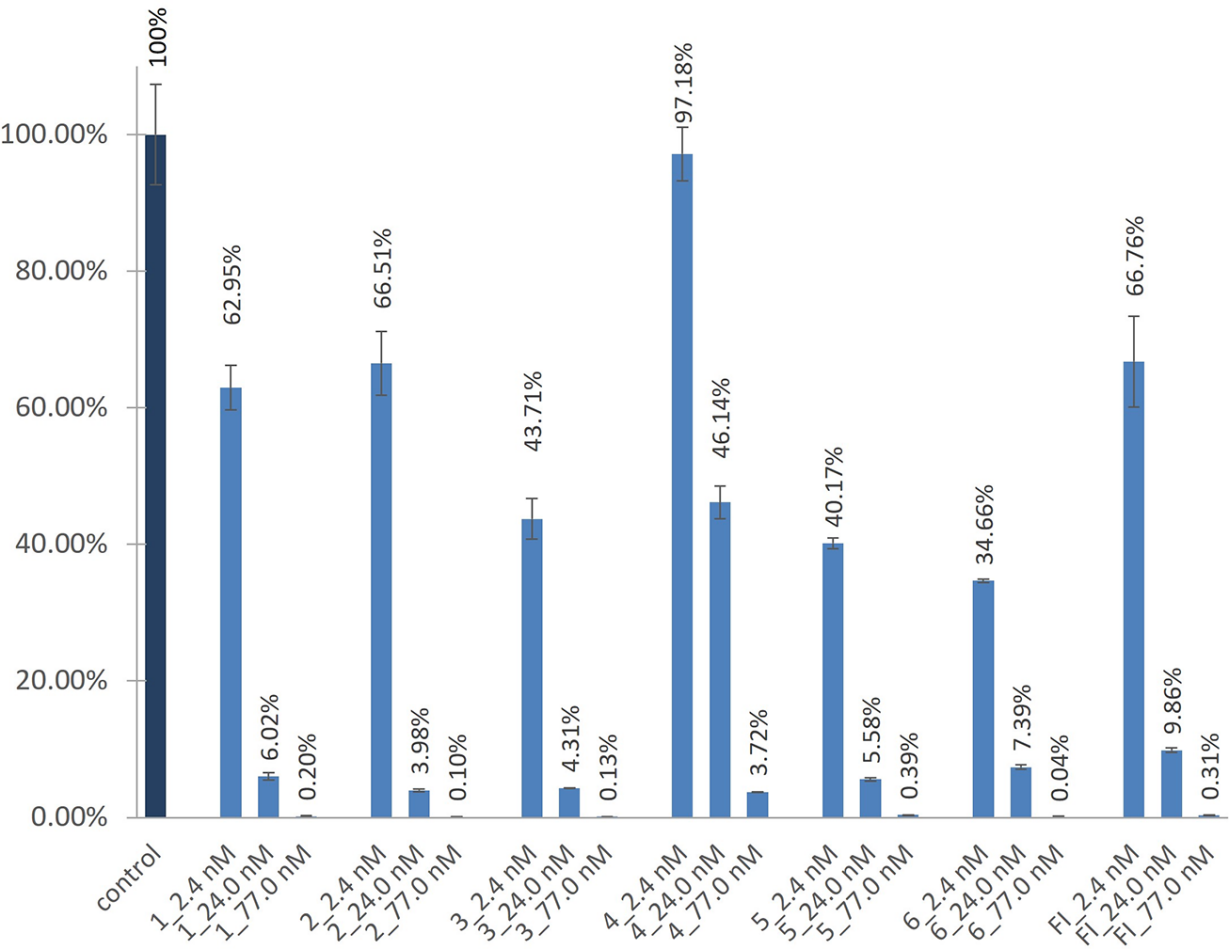
Inhibition of furin versus control with no inhibitor added.

**Table 2:** Inhibitory Properties of Selected Peptides against Furin^a^

inhibitor	IC_50_ [nM]	*K*_i_ [nM]
**3**	3.07 ± 0.20	0.27 ± 0.02
**5**	2.41 ± 0.12	0.21 ± 0.01
**6**	2.91 ± 0.17	0.25 ± 0.01
FI	4.42 ± 0.22	0.38 ± 0.02

a Standard errors of the mean (SEM) are given.

The presence of furin in PANC-1 cell lysate was indicated by analyzing the release of fluorescent AMC product after incubation of furin substrate (Pyr-Arg-Thr-Lys-Arg-AMC) in the lysate, as described elsewhere.^31,32^ However, we are aware that this assay tends to reflect total furin-like enzyme activity rather than the activity of furin exclusively. This is the case because the liberation of AMC may be catalyzed not only by furin but also by an enzyme with a similar substrate preference, such as other PCs.^31^ All selected furin inhibitors (**3**, **6**, **5**, and FI) reduced furin-like activity in cell lysate, with the highest potency presented by FI (about 50% reduction of initial enzyme activity) (Figure 5). For comparison, the effect of a strong trypsin inhibitor, monocyclic SFTI-1, on furin-like activity was insignificant under the same assay conditions. Interestingly, a stronger inhibitory effect was observed when incubation of furin inhibitors in cell lysate was preceded with addition of SFTI-1. It is likely that SFTI-1 may provide protection for furin inhibitors from degradation by trypsin-like proteases.

**Figure 5:**
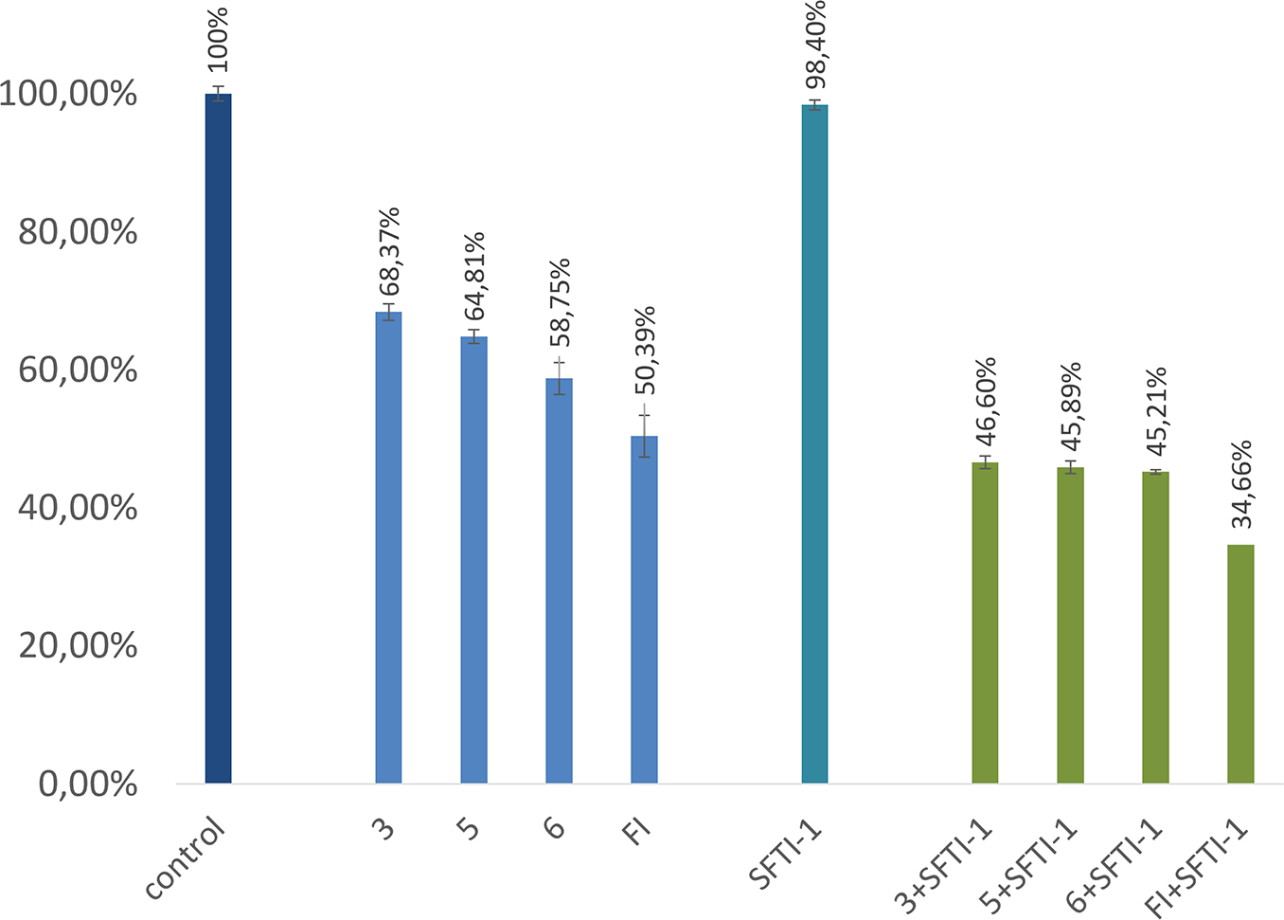
Inhibition of furin-like enzyme activity in PANC-1 cell lysate with or without SFTI-1 addition versus control with no peptide added.

The selectivities of the strongest furin inhibitors **3**, **5**, **6**, and FI were assessed using commercially available, purified, recombinant proteases, namely, matriptase-1 (MT1) and matriptase-2 (MT2). Both enzymes belong to the type II transmembrane serine proteases, present trypsin-like specificity, and share high structural similarity but differ in biological activity.^33^ Recently we showed that SFTI-1 may be regarded as a relevant scaffold to design efficient inhibitors of MT1 and MT2.^34,35^ In this study, inhibitors **3**, **6**, **5**, and FI were not active against MT1 and displayed only irrelevant activity in assay with MT2 (Figure S1), therefore supporting their selectivity toward furin.

Next, we examined the stabilities of inhibitors **3**, **5**, **6**, and FI in human serum (Figure 6). As expected, both monocyclic peptides (**3** and FI) were degraded more rapidly than bicyclic inhibitors **5** and **6**. After 5 h of incubation, about 25% of intact peptide **3** and 15% of FI were detected by HPLC. Both peptides were completely degraded after 24 h, and their MS and HPLC signals were undetected. By contrast, the bicyclic peptides were clearly more resistant and stable. About 59% and 28% (based on HPLC data) of the initial concentrations of peptide **5** and peptide **6**, respectively, remained unchanged even after 48 h of incubation. A closer analysis of MS data revealed the presence of *m*/*z* signals with low intensities that might correspond to the truncated parent peptides deprived of one, two, or three Arg residues (in the case of peptide **6**) and Lys-Arg or Lys-Arg-Arg fragments in the case of peptide **5**. This possible protease-driven degradation was not confirmed in any additional experiments and must be treated with caution. It might be also speculated that the observed decrease in concentrations of both bicyclic peptides is related to binding to serum proteins during the long incubation process and subsequent sample preparation before HPLC analysis, as mentioned elsewhere.^36^ Generally, our results are in accordance with the widely recognized observation that macrocyclization of peptides increases their stability.

**Figure 6:**
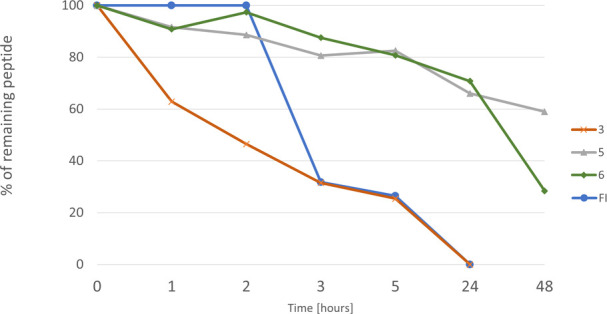
Stabilities of selected peptides in human serum.

Further, some MD simulations were performed to better understand furin–inhibitor interactions. Five repeats of the 100 ns MD simulation of the furin–inhibitor complexes were performed to analyze stability and binding free energies in the studied systems. All furin–inhibitor complexes were stable during the MD runs, and no dissociation event was observed. It can be seen that monocyclic disulfide-bridged inhibitor **3** and its shorter analogue **1** are located deeper in the catalytic pocket than monocyclic inhibitors **2** and FI as well as bicyclic **5**.

The molecular mechanics–generalized Born surface area (MM-GBSA) analysis was performed based on whole trajectories to check which of the studied systems are energetically more favorable. At the same time we applied linear interaction energy (LIE) analysis to compare the data from both approaches (Table 3).

**Table 3:** MM-GBSA and LIE Binding Free Energy Analysis of Furin–Inhibitor Complexes

	Δ*G* [kcal/mol]	
inhibitor	MM-GBSA	LIE
**1**	–87.3 ± 16.6	–73.9 ± 8.2
**2**	–73.2 ± 9.4	–70.6 ± 5.5
**3**	–90.2 ± 7.3	–83.6 ± 3.9
**5**	–54.4 ± 6.9	–53.1 ± 8.1
FI	–46.7 ± 15.7	–54.4 ± 13.3

The results obtained by the MM-GBSA and LIE methods are qualitatively very similar, and the stabilities of interactions in furin–inhibitor complexes estimated on their basis follow the same ranking. The most stable interactions, as reflected in the lowest free energy of binding (Δ*G*), were found for the furin–**3** complex (MM-GBSA = −90.2 ± 7.3 kcal/mol; LIE = 83.6 ± 3.9 kcal/mol). A slightly less favorable Δ*G* value was determined for the furin–**1** complex (MM-GBSA = −87.3 ± 16.6 kcal/mol; LIE = −73.9 ± 8.2 kcal/mol). It is worth noticing that the closely related monocyclic inhibitors **1** and **3** contain two (positions P4 and P5) or three (P4–P6) N-terminal Arg residues, respectively, while other tested inhibitors contain one Lys at either P5 or P6.

For all amino acid residues in furin–inhibitor complexes, decomposed energies per residue were calculated, suggesting which residues in both interacting partners are the most significant for the complex formation (Table S1 and Figure S2).

In the per-residue decomposition analysis of the enzyme, all amino acid residues for which the Δ*G* value was lower than −2.0 kcal/mol are presented. The highest number of enzyme residues with favorable binding contributions was observed in the complexes of furin with inhibitors **3** and **1**. Also, two of three of the catalytic triad amino acid residues, Asp 153 and His 194, are strongly involved in the complex formation in all of the studied complexes. The third residue from the catalytic triad, Ser 368, is present only in the furin–**3** complex, probably due to the fact that this is the most energetically favorable complex. Due to the high structural similarity of the calculated binding poses, similar residues contribute to the binding for all inhibitors (Tables S1 and S2). This binding profile is also very similar to the ones obtained in the literature for inhibitor FI and other substrate analogue inhibitors.^13,26^ Results of the per-residue decomposition for the ligand residues in furin–inhibitor complexes show that for all of the studied systems, inhibitor residues have very similar Δ*G* values (less favorable than −5 kcal/mol) for the middle and C-terminal part of the inhibitors. The largest differences can be found in the N-terminal part, reflecting the most favorable Δ*G* values for inhibitor **1** (Arg 1 and Arg 2). The calculations suggest that inhibitors **1**, **2**, **3**, and **5** have a higher potency than FI.^13^

Interesting differences in Δ*G* values can be seen for disulfide-bridged inhibitor **2** and its bicyclic analogue **5**, which according to the enzymatic assay is the most potent furin inhibitor (*K*_i_ = 0.21 nM). In the N-terminal part, inhibitor **2** has more favorable energies than inhibitor **5**. This is probably due to the fact that inhibitor **2** has greater freedom of movement and thus a better possibility of conformational fit to the receptor corresponding the observed gain in the enthalpy.

In order to check how the individual residues of the inhibitors fit to the enzyme and are stabilized upon binding, root-mean-square deviation (RMSD) analysis was performed. First, the RMSD reflecting the flexibility of these molecules was analyzed in the MD simulations for the unbound inhibitors (Figure S3) and then for the same ligands in furin–inhibitor complexes (Figure S4).

Conformational changes of the inhibitors during unbounded MD simulations were very similar, and the mean RMSD values are below 2 Å for each residue in each inhibitor. All of the structures are very stable due to the presence of a disulfide bridge in each of them (Cys3–Cys11). Surprisingly, no significant differences can be seen between monocyclic inhibitor **2** and its bicyclic counterpart inhibitor **5**. The RMSD medians are almost identical for these two peptides; however, a careful analysis (Figure S3) reveals that many more points are below the RMSD median value for inhibitor **5** than in the case of inhibitor **2** for the residues Lys0 and Phe12, which are involved in the second cyclization. The RMSD changes for the inhibitors residues in the furin–inhibitor MD simulations are substantially different, with RMSD values reaching 20 Å (inhibitor **2**, Phe12). In general, the highest RMSD values can be observed for the furin–FI complex and the smallest changes for the furin–**1** and furin–**3** complexes. Comparing RMSD values for inhibitors **2** and **5**, a strong similarity between these peptides can be observed. Changes in RMSD at the N- and C-terminus appear to be higher for inhibitor **5**, but many points higher than the median for Lys0, Arg1, Arg2, and Phe12 of inhibitor **2** are observed. This potentially suggests that in the furin–**2** complex the ligand had a greater possibility of movement and conformational fit, putatively leading to the essentially higher entropic loss in the process of binding in comparison to inhibitor **5**.

The analysis of stability and interactions in furin–inhibitor complexes was supplemented by hydrogen-bond analysis for the three (two for inhibitor **1** and FI) first N-terminal residues of the inhibitors (Table S2). The furin amino acid residues located near the catalytic pocket were acceptors of the hydrogen bonds. Higher occupancies of the hydrogen bonds are present in the furin–**1** and furin–**3** complexes, while in the furin–FI complex the fewest stabilizing hydrogen bonds are observed. Comparing inhibitors **2** and **5**, some similarities can be seen, but Lys1 of inhibitor **5** is not involved in the formation of hydrogen bonds due to its participation in the cyclization.

The application of a combinatorial chemistry approach resulted in strong, cyclic, peptidic furin inhibitors. They were designed based on the SFTI-1 framework, the widely recognized framework for engineering inhibitors of various, mostly serine, proteases. The library-derived structure of revealed-here inhibitor **1** was highly similar to that of the inhibitor described by Fittler et al. (peptide FI).^13^ These inhibitors are monocyclic, disulfide-bridged peptide amides with different amino acids at the N-terminal P5 position, i.e., Arg or Lys, respectively. Both of them inhibited furin with thoroughly comparable potencies even though computational study indicated markedly lower energy of binding (Δ*G*) of the furin–**1** complex compared with the furin–FI complex. This could be explained by the higher hydrogen-bonding propensity of the Arg side chain (peptide **1**) containing more potential hydrogen-bonding donors than Lys (FI). Extension of peptide **1** by attaching an additional Arg residue at the P6 position resulted in monocyclic peptide **3** and its bicyclic analogue **6**, while the introduction of Lys led to monocyclic peptide **2** and its bicyclic counterpart, peptide **5**. The extended inhibitors **3** (*K*_i_ = 0.27 nM), **5** (*K*_i_ = 0.21 nM), and **6** (*K*_i_ = 0.25 nM) displayed the strongest inhibitory activity among compounds tested in our study. They also revealed inhibitory potency in cell lysate. Surprisingly, monocyclic peptide **2** bearing N-terminal Lys at P6 was less active. The high inhibitory activity of disulfide-bridged peptide **3** was supported by MD simulations. On the other hand, our study suggests that the higher inhibitory activity of bicyclic peptide **5** in comparison to peptide **2** is associated with lower entropic loss in the process of binding of the former peptide with the enzyme. Nonetheless, we demonstrated that a basic amino acid at the P6 position has a beneficial effect on furin inhibition. Our results underpin those presented by Dahms et al.,^26^ who also pointed out the beneficial role of a basic residue at the P6 position. Backbone cyclization of the most potent inhibitors **5** and **6** renders them significantly proteolytically stable in serum.

## Supporting Information Available

MD simulation details, experimental details, materials and methods, and MS and HPLC analyses (PDF)

## Abbreviations

Agp: 2-amino-3-guanidinopropionic acid
AMC: 7-amino-4-methylcoumarin
Cit: citrulline
FI: Fittler inhibitor
Gnf: 4-guanidinophenylalanine
hArg: homoarginine
LIE: linear interaction energy
MD: molecular dynamics
MM-GBSA: molecular mechanics–generalized Born surface area
MT1: matriptase-1
MT2: matriptase-2
Orn: ornithine
PACE: paired basic amino acid cleaving enzyme
Phe(4-NH_2_): 4-aminophenylalanine
Pyr: pyroglutamic acid
RMSD: root-mean-square deviation
SFTI-1: sunflower trypsin inhibitor-1
Xaa: any amino acid

## References

[ref1] Thomas G. Furin at the Cutting Edge: From Protein Traffic to Embryogenesis and Disease. Nat. Rev. Mol. Cell Biol. 2002, 3 (10), 753–766. 10.1038/nrm934. 12360192 PMC1964754

[ref2] Seidah N. G.; Prat A. The Biology and Therapeutic Targeting of the Proprotein Convertases. Nat. Rev. Drug Discovery 2012, 11 (5), 367–383. 10.1038/nrd3699. 22679642

[ref3] Osman E. E. A.; Rehemtulla A.; Neamati N. Why All the Fury over Furin?. J. Med. Chem. 2022, 65 (4), 2747–2784. 10.1021/acs.jmedchem.1c00518. 34340303

[ref4] Furin. The Human Protein Atlas. https://www.proteinatlas.org/ENSG00000140564-FURIN (accessed 2023-01-13).

[ref5] Thomas G.; Couture F.; Kwiatkowska A. The Path to Therapeutic Furin Inhibitors: From Yeast Pheromones to SARS-CoV-2. Int. J. Mol. Sci. 2022, 23 (7), 3435 10.3390/ijms23073435. 35408793 PMC8999023

[ref6] Molloy S. S.; Anderson E. D.; Jean F.; Thomas G. Bi-Cycling the Furin Pathway: From TGN Localization to Pathogen Activation and Embryogenesis. Trends Cell Biol. 1999, 9 (1), 28–35. 10.1016/S0962-8924(98)01382-8. 10087614

[ref7] Braun E.; Sauter D. Furin-Mediated Protein Processing in Infectious Diseases and Cancer. Clin. Transl. Immunol. 2019, 8 (8), 1–19. 10.1002/cti2.1073. PMC6682551 31406574

[ref8] Basak A. Inhibitors of Proprotein Convertases. J. Mol. Med. 2005, 83 (11), 844–855. 10.1007/s00109-005-0710-0. 16215768

[ref9] Couture F.; Kwiatkowska A.; Dory Y. L.; Day R. Therapeutic Uses of Furin and Its Inhibitors: A Patent Review. Expert Opin. Ther. Pat. 2015, 25 (4), 379–396. 10.1517/13543776.2014.1000303. 25563687

[ref10] Lewandowska-Goch M. A.; Kwiatkowska A.; Łepek T.; Ly K.; Navals P.; Gagnon H.; Dory Y. L.; Prahl A.; Day R. Design and Structure-Activity Relationship of a Potent Furin Inhibitor Derived from Influenza Hemagglutinin. ACS Med. Chem. Lett. 2021, 12 (3), 365–372. 10.1021/acsmedchemlett.0c00386. 33738063 PMC7957945

[ref11] Ivanova T.; Hardes K.; Kallis S.; Dahms S. O.; Than M. E.; Künzel S.; Böttcher-Friebertshäuser E.; Lindberg I.; Jiao G. S.; Bartenschlager R.; Steinmetzer T. Optimization of Substrate-Analogue Furin Inhibitors. ChemMedChem 2017, 12 (23), 1953–1968. 10.1002/cmdc.201700596. 29059503

[ref12] Bruno B. J.; Miller G. D.; Lim C. S. Basics and Recent Advances in Peptide and Protein Drug Delivery. Ther. Delivery 2013, 4 (11), 1443–1467. 10.4155/tde.13.104. PMC3956587 24228993

[ref13] Fittler H.; Depp A.; Avrutina O.; Dahms S. O.; Than M. E.; Empting M.; Kolmar H. Engineering a Constrained Peptidic Scaffold towards Potent and Selective Furin Inhibitors. ChemBioChem 2015, 16 (17), 2441–2444. 10.1002/cbic.201500447. 26426719

[ref14] Korsinczky M. L. J.; Schirra H. J.; Rosengren K. J.; West J.; Condie B. A.; Otvos L.; Anderson M. A.; Craik D. J. Solution Structures by 1H NMR of the Novel Cyclic Trypsin Inhibitor SFTI-1 from Sunflower Seeds and an Acyclic Permutant. J. Mol. Biol. 2001, 311 (3), 579–591. 10.1006/jmbi.2001.4887. 11493011

[ref15] de Veer S. J.; White A. M.; Craik D. J. Sunflower Trypsin Inhibitor-1 (SFTI-1): Sowing Seeds in the Fields of Chemistry and Biology. Angew. Chem., Int. Ed. 2021, 60 (15), 8050–8071. 10.1002/anie.202006919. 32621554

[ref16] Houghten R. A.; Pinilla C.; Blondelle S. E.; Appel J. R.; Dooley C. T.; Cuervo J. H. Generation and Use of Synthetic Peptide Combinatorial Libraries for Basic Research and Drug Discovery. Nature 1991, 354, 84–86. 10.1038/354084a0. 1719428

[ref17] Łęgowska A.; Dębowski D.; Lesner A.; Wysocka M.; Rolka K. Selection of Peptomeric Inhibitors of Bovine α-Chymotrypsin and Cathepsin G Based on Trypsin Inhibitor SFTI-1 Using a Combinatorial Chemistry Approach. Mol. Diversity 2010, 14 (1), 51–58. 10.1007/s11030-009-9142-z. 19357983

[ref18] Gruba N.; Wysocka M.; Brzezińska M.; Dębowski D.; Sieńczyk M.; Gorodkiewicz E.; Guszcz T.; Czaplewski C.; Rolka K.; Lesner A. Bladder Cancer Detection Using a Peptide Substrate of the 20S Proteasome. FEBS J. 2016, 283 (2), 2929–2948. 10.1111/febs.13786. 27326540

[ref19] Wysocka M.; Gruba N.; Miecznikowska A.; Popow-Stellmaszyk J.; Gütschow M.; Stirnberg M.; Furtmann N.; Bajorath J.; Lesner A.; Rolka K. Substrate Specificity of Human Matriptase-2. Biochimie 2014, 97 (1), 121–127. 10.1016/j.biochi.2013.10.001. 24161741

[ref20] Zabłotna E.; Jaśkiewicz A.; Łęgowska A.; Miecznikowska H.; Lesner A.; Rolka K. Design of Serine Proteinase Inhibitors by Combinatorial Chemistry Using Trypsin Inhibitor SFTI-1 as a Starting Structure. J. Pept. Sci. 2007, 13 (11), 749–755. 10.1002/psc.887. 17828796

[ref21] Kacprzak M. M.; Peinado J. E.; Than M. E.; Appel J.; Henrich S.; Lipkind G.; Houghten R. A.; Bode W.; Lindberg I. Inhibition of Furin by Polyarginine-Containing Peptides: Nanomolar Inhibition by Nona-d-Arginine. J. Biol. Chem. 2004, 279 (35), 36788–36794. 10.1074/jbc.M400484200. 15197180

[ref22] Cameron A.; Appel J.; Houghten R. A.; Lindberg I. Polyarginines Are Potent Furin Inhibitors. J. Biol. Chem. 2000, 275 (47), 36741–36749. 10.1074/jbc.M003848200. 10958789

[ref23] Fugere M.; Appel J.; Houghten R. A.; Lindberg I.; Day R. Short Polybasic Peptide Sequences Are Potent Inhibitors of PC5/6 and PC7: Use of Positional Scanning-Synthetic Peptide Combinatorial Libraries as a Tool for the Optimization of Inhibitory Sequences. Mol. Pharmacol. 2007, 71 (1), 323–332. 10.1124/mol.106.027946. 17012622

[ref24] Levesque C.; Fugère M.; Kwiatkowska A.; Couture F.; Desjardins R.; Routhier S.; Moussette P.; Prahl A.; Lammek B.; Appel J. R.; Houghten R. A.; D’Anjou F.; Dory Y. L.; Neugebauer W.; Day R. The Multi-Leu Peptide Inhibitor Discriminates between PACE4 and Furin and Exhibits Antiproliferative Effects on Prostate Cancer Cells. J. Med. Chem. 2012, 55 (23), 10501–10511. 10.1021/jm3011178. 23126600 PMC3523546

[ref25] Dooley C. T.; Houghten R. A. The Use of Positional Scanning Synthetic Peptide Combinatorial Libraries for the Rapid Determination of Opioid Receptor Ligands. Life Sci. 1993, 52 (18), 1509–1517. 10.1016/0024-3205(93)90113-H. 8387136

[ref26] Dahms S. O.; Hardes K.; Steinmetzer T.; Than M. E. X-Ray Structures of the Proprotein Convertase Furin Bound with Substrate Analogue Inhibitors Reveal Substrate Specificity Determinants beyond the S4 Pocket. Biochemistry 2018, 57 (6), 925–934. 10.1021/acs.biochem.7b01124. 29314830

[ref27] Spengler J.; Jiménez J. C.; Burger K.; Giralt E.; Albericio F. Abbreviated Nomenclature for Cyclic and Branched Homo- and Hetero-Detic Peptides. J. Pept. Res. 2005, 65 (6), 550–555. 10.1111/j.1399-3011.2005.00254.x. 15885114

[ref28] Ramos-Molina B.; Lick A. N.; Nasrolahi Shirazi A.; Oh D.; Tiwari R.; El-Sayed N. S.; Parang K.; Lindberg I. Cationic Cell-Penetrating Peptides Are Potent Furin Inhibitors. PLoS One 2015, 10 (6), e0130417 10.1371/journal.pone.0130417. 26110264 PMC4482483

[ref29] Łepek T.; Kwiatkowska A.; Couture F.; Ly K.; Desjardins R.; Dory Y.; Prahl A.; Day R. Macrocyclization of a Potent PACE4 Inhibitor: Benefits and Limitations. Eur. J. Cell Biol. 2017, 96 (5), 476–485. 10.1016/j.ejcb.2017.04.001. 28483279

[ref30] Van Lam van T.; Ivanova T.; Hardes K.; Heindl M. R.; Morty R. E.; Böttcher-Friebertshäuser E.; Lindberg I.; Than M. E.; Dahms S. O.; Steinmetzer T. Design, Synthesis, and Characterization of Macrocyclic Inhibitors of the Proprotein Convertase Furin. ChemMedChem 2019, 14 (6), 673–685. 10.1002/cmdc.201800807. 30680958

[ref31] Bourne G. L.; Grainger D. J. Development and Characterisation of an Assay for Furin Activity. J. Immunol. Methods 2011, 364 (1–2), 101–108. 10.1016/j.jim.2010.11.008. 21112328

[ref32] Sawada Y.; Inoue M.; Kanda T.; Sakamaki T.; Tanaka S.; Minamino N.; Nagai R.; Takeuchi T. Co-Elevation of Brain Natriuretic Peptide and Proprotein-Processing Endoprotease Furin after Myocardial Infarction in Rats. FEBS Lett. 1997, 400 (2), 177–182. 10.1016/S0014-5793(96)01385-3. 9001393

[ref33] Bugge T. H.; Antalis T. M.; Wu Q. Type II Transmembrane Serine Proteases. J. Biol. Chem. 2009, 284 (35), 23177–23181. 10.1074/jbc.R109.021006. 19487698 PMC2749090

[ref34] Gitlin-Domagalska A.; Dębowski D.; Łęgowska A.; Stirnberg M.; Okońska J.; Gütschow M.; Rolka K. Design and Chemical Syntheses of Potent Matriptase-2 Inhibitors Based on Trypsin Inhibitor SFTI-1 Isolated from Sunflower Seeds. Biopolymers 2017, 108 (6), e23031 10.1002/bip.23031. 28555756

[ref35] Gitlin A.; Dębowski D.; Karna N.; Łęgowska A.; Stirnberg M.; Gütschow M.; Rolka K. Inhibitors of Matriptase-2 Based on the Trypsin Inhibitor SFTI-1. ChemBioChem 2015, 16 (11), 1601–1607. 10.1002/cbic.201500200. 25999208

[ref36] Chan L. Y.; Gunasekera S.; Henriques S. T.; Worth N. F.; Le S. J.; Clark R. J.; Campbell J. H.; Craik D. J.; Daly N. L. Engineering Pro-Angiogenic Peptides Using Stable, Disulfide-Rich Cyclic Scaffolds. Blood 2011, 118 (25), 6709–6717. 10.1182/blood-2011-06-359141. 22039263

