# Supplementary material for: Cyclic Peptidic Furin Inhibitors Developed by Combinatorial Chemistry
Source: ACS Med Chem Lett. 2023 Mar 15;14(4):458–65. doi: 10.1021/acsmedchemlett.3c00008 (PMC10107917; doi:10.1021/acsmedchemlett.3c00008)
Supplement: Supplementary file 1 — ml3c00008_si_001.pdf [file ml3c00008_si_001.pdf]

Cyclic peptidic furin inhibitors developed by combinatorial chemistry

Agata Gitlin-Domagalska[1], Dawid Dębowski[1]*, Aleksandra Maciejewska[1], Sergey Samsonov[2], Martyna Maszota-Zieleniak[2], Natalia Ptaszyńska[1], Anna Łęgowska[1], Krzysztof Rolka[1]

[1]Departement of Molecular Biochemistry, Faculty of Chemistry, University of Gdansk

[2] Department of Theoretical Chemistry, Faculty of Chemistry, University of Gdansk

Inhibition of matriptase-1 and matriptase-2 ....S3
Molecular dynamics simulations S3
Experimental section S6
Chemical synthesis and deconvolution of peptide library..S6
Resynthesis of the selected inhibitors and purification..S7
N-Fmoc-4-(tert-butoxycarbonyl)amino-L-phenylalanine synthesis..S8
Enzymatic assays..S8
Total furin-like enzyme inhibition/activity in PANC-1 cell lysates..S9
Stability in serum..S9
Molecular dynamics simulations..S10
HPLC chromatograms of synthesized peptides..S11
MS analyses of synthesized peptides..S20
N-Fmoc-4-(tert-butoxycarbonyl)amino-L-phenylalanine MS and HPL analyses S23
Serum stability HPLC chromatograms S25
References..S26

List of figures:

Figure S1. Study of selected inhibitors against matriptase-1 and matriptase-2. (S2)

Figures S2-S5. Molecular dynamics simulations. (S4-S11)

Figures S6-S19. HPLC Chromatograms of synthesized inhibitors 1-6 and FI. (S12-S19)

Figures S20-S26. MS analyses of synthesized inhibitors 1-6 and FI. (S20-S27)

Figures S27-S28. HPLC and MS analyses of N-Fmoc-4-(tert-butoxycarbonyl)amino-L-phenylalanine. (S23-S24)

Figure S29. Serum stability HPLC chromatograms. (S25)

List of tables:

Table S1. MM-GBSA per residue decomposition of furin-inhibitor complexes. (S3)

Table S2. Hydrogen bonds in furin-inhibitor complexes during MD simulations. (S5-S6)

Inhibition of matriptase-1 and matriptase-2

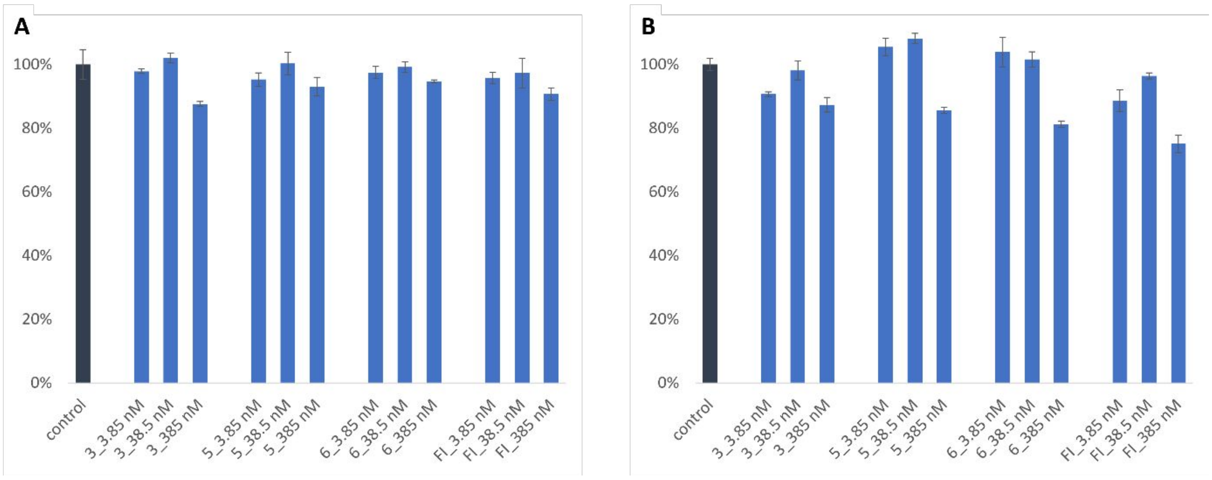

Figure S1. Study of selected inhibitors against matriptase-1 (A) and matriptase-2 (B). Control presents activity of an enzyme with no peptide added.

Molecular dynamics simulations

Table S1S. MM-GBSA per residue decomposition of furin-inhibitor complexes.

Furin-inhibitor									
1		2		3		5		FI	
residue	ΔG [kcal/mol]	residue	ΔG [kcal/mol]	residue	ΔG [kcal/mol]	residue	ΔG [kcal/mol]	residue	ΔG [kcal/mol]
Asp154	-4.5	His194	-4.2	Trp254	-4.0	Asp153	-3.5	Asp154	-4.3
Glu257	-4.3	Asp228	-3.6	His194	-3.7	His194	-3.0	His194	-2.9
Asp153	-4.1	Asp154	-3.4	Glu236	-3.4	Ser255	-2.4	Asp228	-2.3
Trp254	-3.2	Asp153	-2.6	Ser253	-3.4			Asp191	-2.1
Ala292	-2.6	Glu230	-2.6	Asp306	-3.2			Asp153	-2.0
Asp306	-2.6	Ser253	-2.5	Leu227	-2.9				
His194	-2.3	Asp191	-2.1	Asp153	-2.5				
Glu236	-2.3	Thr365	-2.1	Ala292	-2.4				
Leu227	-2.2			Glu257	-2.1				
				Ser368	-2.0				

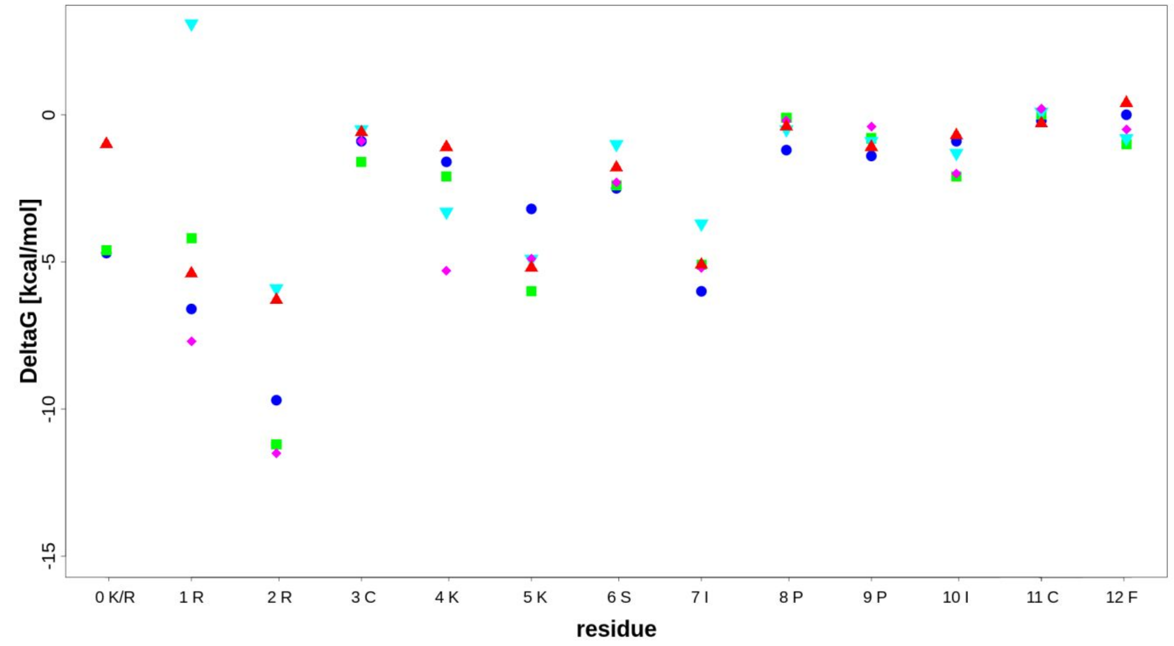

Figure S2. Per residue decomposition analysis for the ligand residues in furin-inhibitor complexes (inhibitors – 1 magenta rhombus, 2 blue circle, 3 green square , 5 red triangle and FI cyan inverted triangle). Residues are numbered from zero so that sequential similarity is preserved regardless of inhibitor length (inhibitors 1 and FI has one residue less in N-terminal part).

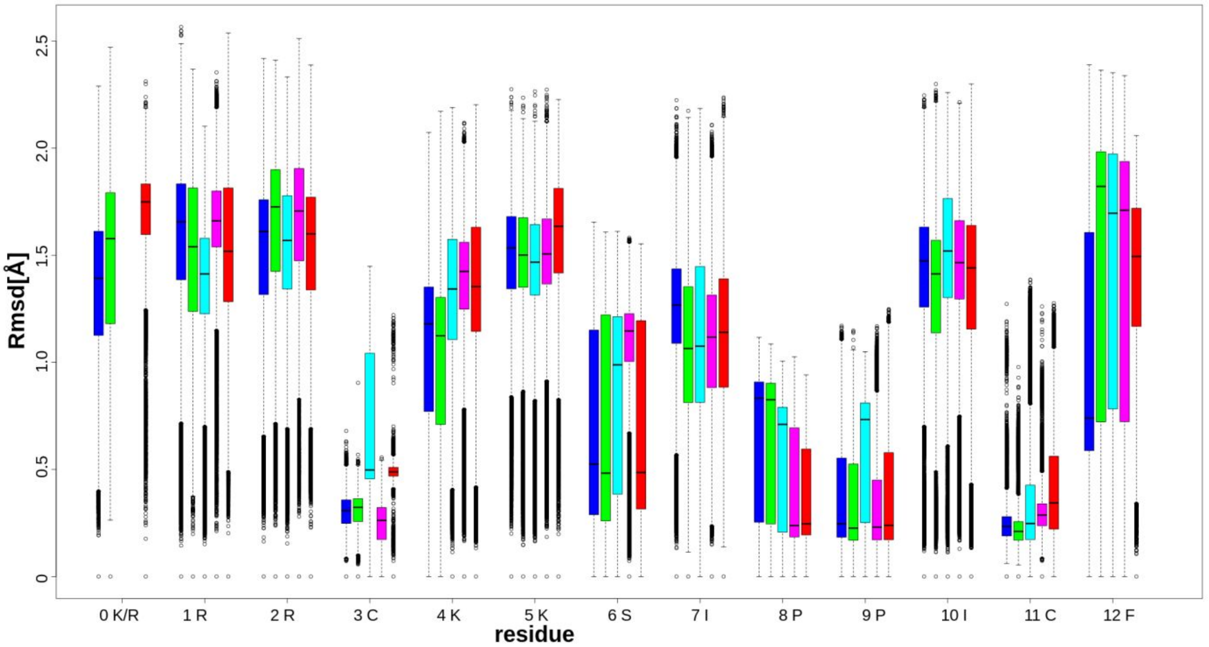

Figure S3. RMSD boxplots for the inhibitor residues in the unbounded MD simulations (inhibitors – 1 magenta, 2 blue, 3 green, 5 red and FI cyan). Residues are numbered from zero so that sequential similarity is preserved regardless of inhibitors length (inhibitor 1 and FI have one residue less in N-terminal part).

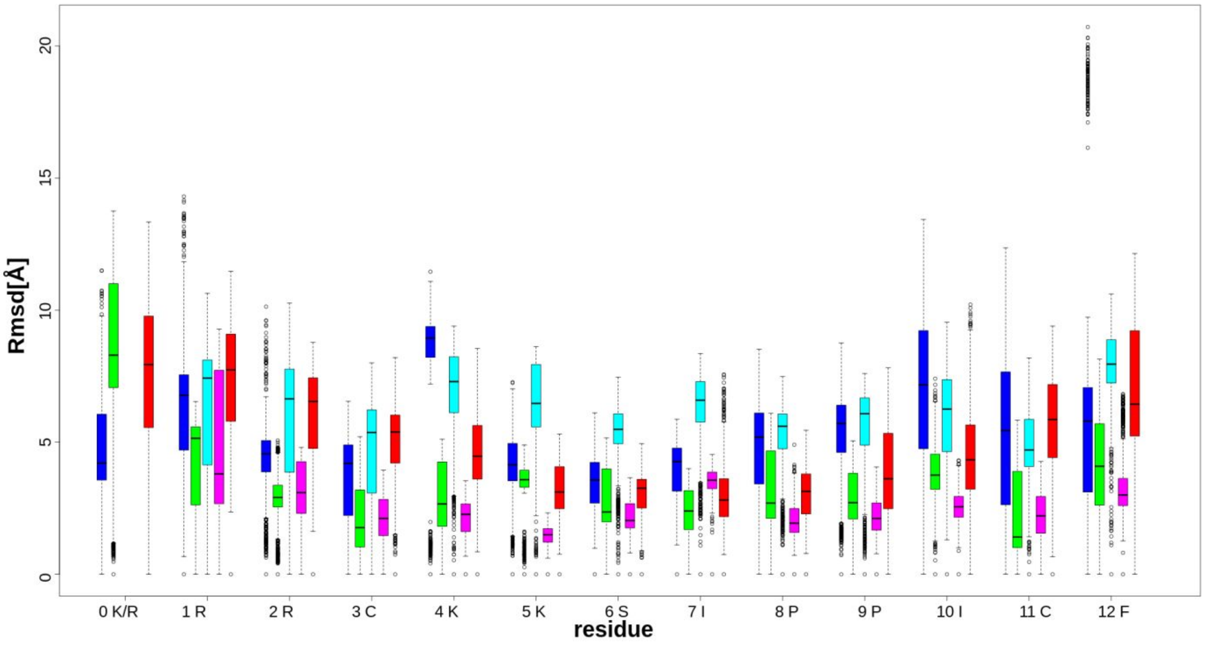

Figure S4. RMSD boxplots for the inhibitor residues in the furin-inhibitor complexes MD simulations (inhibitors – 1 magenta, 2 blue, 3 green, 5 red and FI cyan). Residues are numbered from zero so that sequential similarity is preserved regardless of inhibitor length (inhibitors 1 and FI have one residue less in N-terminal part).

Table S2. Hydrogen bonds in furin-inhibitor complexes during MD simulations (occupancies higher than 5% are considered).

**furin-inhibitor 1**		
acceptor (furin)	donor (inhibitor)	Occupancy [%]
Glu 257	Arg 1	96
Glu 236	Arg 2	87
Glu 257	Arg 2	51
Glu 230	Arg 2	42
Gly 255	Arg 2	42
Glu 230	Arg 1	36
Asp 259	Arg 1	35
Asp 258	Arg 1	28
Leu 227	Arg 2	13
Pro 256	Arg 2	7
**furin-inhibitor 2**		
acceptor (furin)	donor (inhibitor)	Occupancy [%]
Glu 257	Arg 3	77
Glu 230	Arg 3	47
Asp 228	Arg 2	45
Asp 154	Arg 2	38
Glu 230	Arg 2	31
Asp 258	Arg 3	29
Asp 258	Lys 1	20
Glu 230	Lys 1	16
Gly 147	Arg 3	14
Pro 148	Arg 3	8
**furin-inhibitor 3**		
acceptor (furin)	donor (inhibitor)	Occupancy [%]
Glu 236	Arg 3	172*

Glu 230	Arg 1	77
Asp 264	Arg 1	43
Glu 257	Arg 1	42
Gly 255	Arg 3	19
Gly 229	Arg 2	17
Asp 258	Arg 2	15
Glu 257	Arg 3	13
Gly 229	Arg 1	11
Glu 230	Arg 2	6
Glu 257	Arg 2	6
**furin-inhibitor 5**		
acceptor (furin)	donor (inhibitor)	Occupancy [%]
Glu 230	Arg 3	41
Glu 257	Arg 2	36
Glu 230	Arg 2	35
Glu 236	Arg 3	30
Asp 259	Arg 2	18
Leu 227	Arg 3	18
Thr 262	Arg 2	13
Asp 258	Arg 3	7
**furin- FI**		
acceptor (furin)	donor (inhibitor)	Occupancy [%]
Glu 230	Arg 2	54
Glu 257	Arg 2	23
Asp 259	Arg 2	18
Glu 230	Lys 1	17
Val 231	Lys 1	10
Asp 191	Arg 2	5
Glu 257	Lys 1	5

**Values of a hydrogen bond occupancy higher than 100 % correspond to the bifurcated H-bonds or originate from different atomic groups of the same residues participating in hydrogen bond formation.

Experimental section

No unexpected or unusually high safety hazards were encountered.

Chemical synthesis and deconvolution of peptide library

The chemical synthesis of peptide library was carried out using the portioning–mixing (known also as **split and mix**) method. Its deconvolution was performed by the iterative method in solution.[1] Initially, 4 g of the Rink Amide MBHA (loading 0,4 mmol/g) was used. A combinatorial library of SFTI–1 analogues was be obtained with the general formula:

Xaa_l-Xaa_k-Cys(&)-Xaa_j-Xaa_i-Ser-Ile-Pro-Pro-Ile-Cys(&)-Phe-NH_2

and deconvoluted in the following stages:

1. *C*–Terminal heptapeptide amide was synthesised manually by SPPS using Fmoc strategy. After completed coupling of Fmoc-Ser and subsequent Fmoc removal, the peptidyl resin was divided into 2 (*i*) equal portions. One portion was acylated with Fmoc-Lys(Boc) and the other with Fmoc-Arg(Pbf). Each aa derivative coupling was repeated twice, with 3 eq applied for the first coupling and 1.5 eq in case of the second coupling. In the case of Fmoc-Agp($Boc)_2$ and Fmoc-

Phe(4-NH-Boc) we experienced difficulties with complete coupling. So we performed reactions by applying microwave heating (50 °C) which improved the process. The same procedures were applied during the whole library synthesis. 10% of mass of each portion was removed for deconvolution.

2. After that, both portions were combined and divided into 10 (*j*) equal portions, each of which was acylated individually with different protected amino acid. 10% of the mass of each portion was removed for deconvolution.
3. After mixing all *j* portions of peptidyl–resin, Cys protected derivative was attached.
4. Step 2 (splitting, acylation, sample removal, mixing) was repeated three time (splitting into *k*, *l* portions, respectively, *k* and *l* equal 10).
5. Sublibraries (*l* portions of peptidyl-resin) were cleaved from the solid support together with all protecting groups (described below), and subjected to the deconvolution in the iterative approach adopting conditions describe in *Screening of the peptide library for furin inhibitors* section.
6. Sublibraries with fixed best hits in Xaa (*l-j*) were subsequently synthesized (using previously put aside 10% of synthesized sublibraries) and their inhibitory activity was determined until the position *Xi*. Number of compounds in the library $\boldsymbol{i \times j \times k \times l = 2 \times 10 \times 10 \times 10 = \underline{2000}}$

Screening of the synthesized peptide library for furin inhibitors (deconvolution) was performed by the iterative method in solution. The inhibitory activity of sublibraries against furin were measured using Pyr-Arg-Thr-Lys-Arg-AMC fluorogenic substrate (Pepta Nova). The experiments were performed on 96-well plates (BRAND, Germany) and microplate reader FluoStar Omega (BMG Labtech, Germany).

In general experimental procedure was as followed: assay buffer (100 mM HEPES with 1 mM $CaCl_2$ and 1.8 mg/mL bovine serum albumin, pH 7.5) was warmed up to 37 °C, then furin was added, followed by an addition of 2.2 ng/mL solution of each peptide sublibrary. After 30 min incubation at 37 °C, the fluorogenic substrate was added. Percentage inhibition of furin was calculated relative to the control sample without inhibitor. In the case of similar results obtained for different sublibraries, the experiments were repeated applying more concentrations of sublibraries.

Resynthesis of the selected inhibitors and purification

Peptides were synthesized using standard 9-fluorenylmethoxycarbonyl (Fmoc) chemistry with a Prelude Peptide Synthesizer (Protein Technology, Inc., USA). Each peptide was synthesized on either Rink Amide resin (loading 0.646 mmol/g, GL Biochem Shanghai) or 2-chlorotrityl chloride resin (Cl atoms 1.46 mmol/g, GL Biochem Shanghai) to produce *C*-terminal amide or *C*-terminal carboxyl groups, respectively. The protected derivatives of all Fmoc-amino acids were purchased from different commercial sources. The peptide chain was elongated in the consecutive cycles of deprotection and coupling. Deprotection was performed with 20% piperidine in *N*,*N*-dimethylformamide (DMF), and peptide chains elongation was performed using a 3-fold molar excess of each Fmoc-protected amino acid, *N*,*N*,*N*,*N'*-tetramethyl-O-(benzotriazol-1yl)-uroniumtetrafluoroborate(TBTU)/1-hydroxybenzotriazole (HOBt)/*N*-methylmorpholine (NMM) (molecular ratio 1:1:1:2). The *C*-terminal amino acid residue was attached to the 2-chlorotrityl chloride resin in the presence of an equimolar amount of *N,N*-diisopropylethylamine (DIPEA) in anhydrous dichloromethane (DCM). In the case of monocyclic peptides, after completing the synthesis, peptides were cleaved from the resin and the protecting groups were removed in a one-step procedure using a mixture of TFA:phenol:triisopropylsilane:H_2O (88:5:2:5, v/v/v/v). Subsequently, the disulfide bridge was formed using a 0.1 M methanolic iodine solution. Bicyclic peptides **1**, **6** and **7** were obtained as described in our previous article.[2] The crude cyclic peptides were purified by reverse-phase high performance liquid

chromatography (RP-HPLC) on PLC 2050 Gilson HPLC with Gilson Glider Prep. Software (Gilson, France), equipped with Grace Vydac C18 (218TP) HPLC column (22 × 250 mm, 10 μm, 300 Å, Resolution Systems). The solvent systems were 0.1% TFA (A) and 80% acetonitrile in A (B). The purity of each peptide was checked by RP-HPLC on a Shimadzu Prominence-I LC-2050C 3D equipped with a Kinetex XB-C18 column (150 × 4.6 mm, 5 μm) and a UV-Vis detector. A linear gradient from 10% to 90% B for 20 min, flow rate 1 mL min^{-1}, and detection at 214 nm was used. The mass spectrometry analysis of the synthesized compounds was carried out on MALDI MS (Autoflex maX MALDI-TOF spectrometer, Bruker Daltonics, Germany) using an α-cyano-4-hydroxycinnamic acid and/or 2,5-dihydroxybenzoic acid matrix.

The pure peptides were obtained with the yields ranging from 62% to 77% in the case of monocyclic, disulfide bridged compounds and 45% to 65% in the case of bicyclic peptides. This calculation includes chemical synthesis, cyclization (disulfide bridge formation and/or 'head to tail' cyclization) and an HPLC purification procedure.

N-Fmoc-4-(tert-butoxycarbonyl)amino-L-phenylalanine synthesis

The introduction of side chain protection on the commercially available Fmoc-Phe(4-NH_2) was performed according to Ozturk et al.[3] In short, 1.5 g Fmoc-Phe(4-NH_2) was dissolved in 200 mL of mixture of dioxane/water (1:1). Then 2.5 eq of $NaHCO_3$ in 10 mL water and 2 eq of Boc_2O in 10 mL dioxane were added to the solution. The reaction mixture was stirred for 24h and then half of the solvent was removed under vacuum evaporator. Reaction progress was monitored using RP-HPLC. Product was extracted into EtOAc (3 × 10 mL). The combined organic extracts were washed with water and then dried over anhydrous $MgSO_4$ and concentrated *in vacuo*. The crude mixture was purified by RP-HPLC. In result 1.3 g of pure product (67% yield) was obtained.

Enzymatic assays

The IC_{50} values (inhibitor concentrations giving 50% inhibition of enzyme activity) were calculated from plots of enzyme activity (% of control sample) *versus* the inhibitor's concentration using a four-parameter fit model (GraFit 5.0.12 Erithacus Software Ltd.). The IC_{50} values were determined from triplicate measurements with at least seven different inhibitor concentrations. The K_i values (Table 1) were calculated using the Cheng-Prusoff equation, assuming the competitive mode of inhibition, according to the following relationship: $IC_{50} = K_i(1+S/K_m)$. All measurements were performed using a Fluostar Omega microplate reader (BMG Labtech, Germany) and 96-well black plates (BRAND, Germany). Recombinant human matriptase-1/ST14 catalytic domain was purchased from R&D Systems, USA. Recombinant human matriptase-2 was obtained from Enzo Life Sciences, Switzerland. Soluble human recombinant furin was kindly gifted by Dr Anna Kwiatkowska (group of Prof. Robert Day from Université de Sherbrooke).[4]

The following assay buffers were used: 50 mM Tris-Cl; pH 8.3 with 150 mM NaCl and 0.01% triton X-100, (matriptase-1 and matriptase-2), pH 7.5 100 mM HEPES with 1 mM $CaCl_2$ and 1.8 mg/mL BSA (furin). The substrates used in the assays were as follows: for matriptase-1 and matriptase-2 Boc-Gln-Ala-Arg-AMC (Pepta Nova, Germany) in final concentration of 5 μM, for furin Pyr-Arg-Thr-Arg-AMC (Pepta Nova, Germany) in final concentration of 20 μM. Enzymes concentrations were as follows: 1.25 ng/mL of matriptase-1, 1.51 U/mL for matriptase-2, and 1.1 nM for furin. In the case of matriptase-1 and matripase-2 concentrations were calculated according to information provided by the suppliers.

In short, the tested compounds were added to the appropriate protease in different concentrations and incubated in an assay buffer at 37 °C for 30 min. After this time, a solution of appropriate substrate was added. Reactions were monitored for at least 40 min at 37 °C. The final volume in each well was 200 μL for matriptases and 100 μL for furin. Measurements were carried out using excitation and emission wavelengths of 380 nm and 450 nm. The percentage inhibition of an enzyme was calculated relative to the control sample without an inhibitor. The K_m value for furin substrate was determined by incubating the enzyme with its substrate in various concentrations as recommended.[5] Plots of the substrate concentration *versus* the initial reaction velocity were analyzed according to Michaelis-Menten equation using GraFit 5.0.12 Erithacus Software Ltd. A double reciprocal (Lineweaver-Burk) plot was used to determine the Michaelis constant K_m value. K_m value determined for furin was 1.90 μM.

Total furin-like enzyme inhibition/activity in PANC-1 cell lysates

Cell line and cell culture. The pancreatic cancer cell line, PANC-1, was obtained from the American Type Culture Collection, ATCC (Manassas, USA). PANC-1, a human pancreatic cancer cell line, was isolated from the pancreatic duct (ATCC, CRL-1469) and was cultured in Dulbecco's Modified Eagle's Medium (DMEM) by supplementing 10% fetal bovine serum (FBS) and 1% of penicillin/streptomycin (P/S). Cells were cultured at 37 °C with 5% CO_2 in a humidified atmosphere. Cells were maintained in a 75 cm^2 tissue culture flask. The medium was replaced every two days. The subcultivation ratio was 1:3 for PANC-1.

Preparation of lysates. PANC-1 cells were seeded in a Petri dishes. After 24 hours, cell lysates were collected and centrifuged at 975 × g at 4 ˚C for 9 minutes. The supernatant was removed, and the cells were resuspended in a cold PBS buffer. The cells were centrifuged again at 900 × g at 4 ˚C for 5 minutes. The supernatant was removed. Cells were suspended in Halt Protease Inhibitor Cocktail, EDTA-Free (100X) (Thermo Fisher Scientific). The samples were stored for 2 hours at -80˚C. The samples were then defrosted and centrifuged at 16 000 × g at 4 ˚C for 20 minutes. The supernatant was stored at -80 ˚C.

The inhibitory potency of selected peptides was examined in cell lysate alone or in presence of sunflower trypsin inhibitor (SFTI-1)[6] sing furin fluorogenic substrate Pyr-Arg-Thr-Arg-AMC (Pepta Nova, Germany). Furin activity was determined by monitoring the AMC liberation over time with a plate reader. Measurements were performed using 96-well black plates (BRAND, Germany).
The general procedure was according to.[7] In short: 20 μL of cell lysate (diluted 10 times with assay buffer pH 7.5 100 mM HEPES with 1 mM $CaCl_2$) was added to the well containing 60 μL of water, after 15 min in 37 °C 10 μL of peptide solution (final concentration in well 100 μM) was added. After 30 min incubation in 37 °C 10 μL substrate (100 μM final concentration) was added.
In the case of experiment performed in presence of SFTI-1 procedure was analogical. Cell lysate was mixed with 50 μL of water, warmed to 37 °C, and then 10 μL of SFTI-1 (100 μM final concentration) was added and incubated for 10 min in 37 °C. Next step was addition of appropriate peptide solution (final concentration in well 100 μM) and 30 min incubation in 37 °C. After this time 10 μL substrate (100 μM final concentration) was added to the well. Percentage inhibition of the enzyme was calculated relative to the control sample without inhibitor.

Stability in serum
Human serum from plasma (Sigma Aldrich, Germany) was prepared for the assay as referred previously.[8] The experiments were carried out according to the procedure described before.[2] The initial concentration of the peptide solutions was 1.54 mM. Each peptide solution was then diluted 10 times with human serum and incubated at 37 °C. Immediately after the start of incubation, 1, 2, 3, 5 and 24

hours the samples of 200 μL were taken and mixed with 200 μL of acetonitrile to separate the human serum proteins. The formed precipitate was removed by centrifugation for 10 min at 13,000 rpm. The supernatant was stored in 3-4º C for at least 1 hour before being analyzed by RP-HPLC (linear gradient 10-90% phase B, 40 min., Kinetex 5 μm XB-C18 100Å 150 x 4.6 mm) and MALDI-MS (Autoflex maX MALDI-TOF spectrometer, Bruker Daltonics, Germany). In order to determine peptide stability at each time point, the peptide peak area from recorded chromatograms was compared with the peptide peak area obtained directly after mixing peptide and plasma, and expressed as its percentage.

Molecular dynamics simulations

The experimental structure of furin was obtained from the PDB (PDB ID: 1P8J 2.6 Å). Furin inhibitors (Figure 5SA) bound to the furin active site were built based on the crystal structure of MT-SP1 in complex with SFTI-1 (PDB ID: 1P8J 2.0 Å) by aligning catalytic triad of both proteins in PyMOL, Molecular Graphics System, Version 2.0 Schrödinger, LLC software. Next, SFTI-1 peptide was replaced by one of the five designed ligands (Figure 5SB). Five resulting furin-inhibitor complexes were further used as starting structures for the molecular dynamic (MD) calculations.

The AMBER20 suite was used to perform MD simulations,[9] ff14SBonlysc[10] force field parameters were used. The initial complex structures were built in the xLeap module of AMBER20. The periodic boundary conditions with TIP3P[11] octahedral water box and Na^+ counterions were used. Two steps of energy minimization were performed: first 500 steepest descent and then 1000 conjugate gradient cycles with harmonic restraints of 10 kcal/mol/$Å^2$ on the solute, next 3000 steepest descent and 3000 conjugate gradient cycles without restraints. Next, the systems were heated up to 300K for 10 ps in NVT ensemble followed by the simulations in NPT ensemble at the pressure of 10^5 Pa. Finally, five independent MD production runs of 100 ns for each furin-inhibitor complex were performed in NPT ensemble. The time integration step was 2 fs, the cutoff for non-bonded interactions was 8 Å, the SHAKE algorithm for the covalent bonds that included hydrogen atoms[12] and the Particle Mesh Ewald procedure[13] were used. The similar MD protocol was applied for systems containing only inhibitor molecules, but production runs were 1 μs for each system.

Energetic post-processing of the trajectories and per residue energy decomposition were done using MM-GBSA with igb=2 for furin-inhibitor complexes. The entropic contribution to binding was not considered since taking into account entropy would increase the overall uncertainty in the calculated binding energies.[14] Therefore, the calculated 'free energies' should be understood as enthalpies which also implicitly partially account for the entropic component of the solvent.

Linear interaction energy (LIE) with a dielectric constant of 80, the analysis of RMSD (root mean squared deviation) and hydrogen bonds established between furin and inhibitors was performed on the MD trajectories using CPPTRAJ Software.[15] All the frames from MD simulations were analyzed. Hydrogen bonds were defined by a hydrogen bond distance cutoff of 3.0 Å and a hydrogen bond angle cutoff of 135°. The number of reported hydrogen bonds was averaged over the entire MD trajectories.

Statistical analysis and visualization. Statistical analysis and graphical presentation of the obtained data were performed by R-package (RC Team, 2013). Each trajectory was visualized in VMD[16] and PyMOL. The PyMOL, was also used for the production of figures.

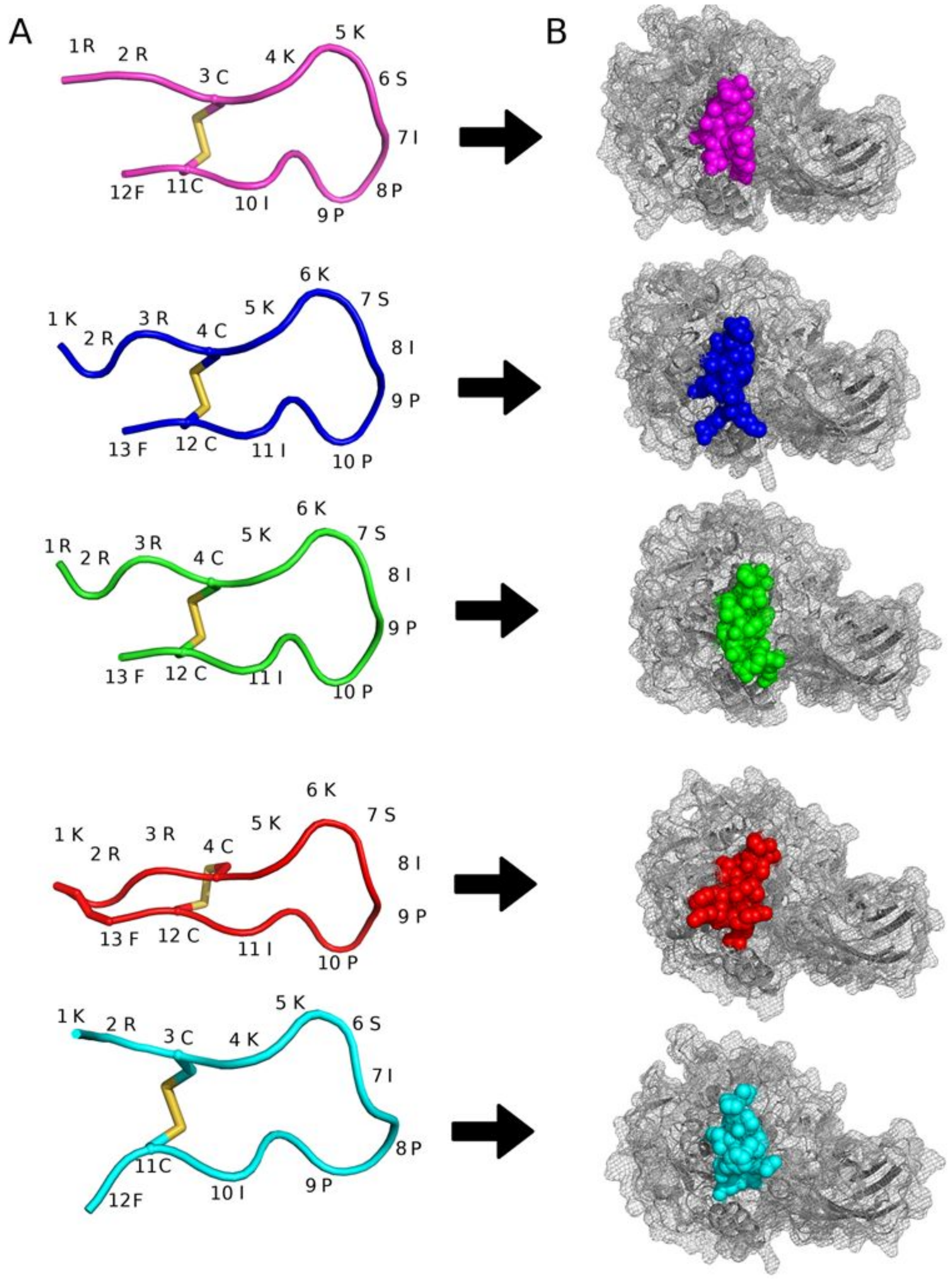

Figure S5. Structures and sequences of the five furin inhibitors (cartoon representation with bridges in licorice representation) (A). Furin-inhibitor docking poses further analyzed with the MD simulations (furin – grey colour, cartoon and mesh representation; inhibitors – 1 magenta, 2 blue, 3 green, 5 red and FI cyan, sphere representation)(B).

HPLC chromatograms of synthesized peptides

HPLC conditions: Shimadzu Prominence-I LC-2050C 3D equipped with a Kinetex XB-C18 column (150 × 4.6 mm, 5 µm) and a UV-Vis detector, linear gradient from 10% to 90% B for 20 min, flow rate 1 mL min−1, detection at 214 nm, column temperature 30ºC or 50ºC.

In contrast to chromatograms recorded at a temperature of 30ºC, showing single signals for bicyclic peptides **4**, **5** and **6**, chromatograms for all monocyclic, disulfide-bridged inhibitors display two, partially merged signals, which suggests the existence of two unresolved compounds.Significantly, in all cases MS analyses provide one signal which confirms the presence of the desired peptide. Despite our tremendous efforts these signals could not be separated by semi-preparative HPLC. A similar observation was made by Fittler et. al. To explain this result, they undermined the quality of used arginine building block. However, in our opinion, isomerization is the most likely explanation of the observed peak splitting. Inhibitors presented in our work contain two proline residues, and it is known that such peptides may exist in both *cis* and *trans* conformations.[17,18] Similar results we obtained for some other proline-containing SFTI-1 analogues (also including *N*-methylated peptides which are considered as highly prone to such isomerization, unpublished data). Moreover, it turned out that HPLC analyses made at higher temperature (50ºC, shown below) resulted in narrow single peaks. Such an observation applies also to bicyclic peptides (**4**, **5**, and **6**). At higher temperature cis-trans interconversion is faster than at 30ºC and signal splitting is not observed. To confirm our speculation, further, more detailed analysis including NMR spectroscopy, will be conducted.

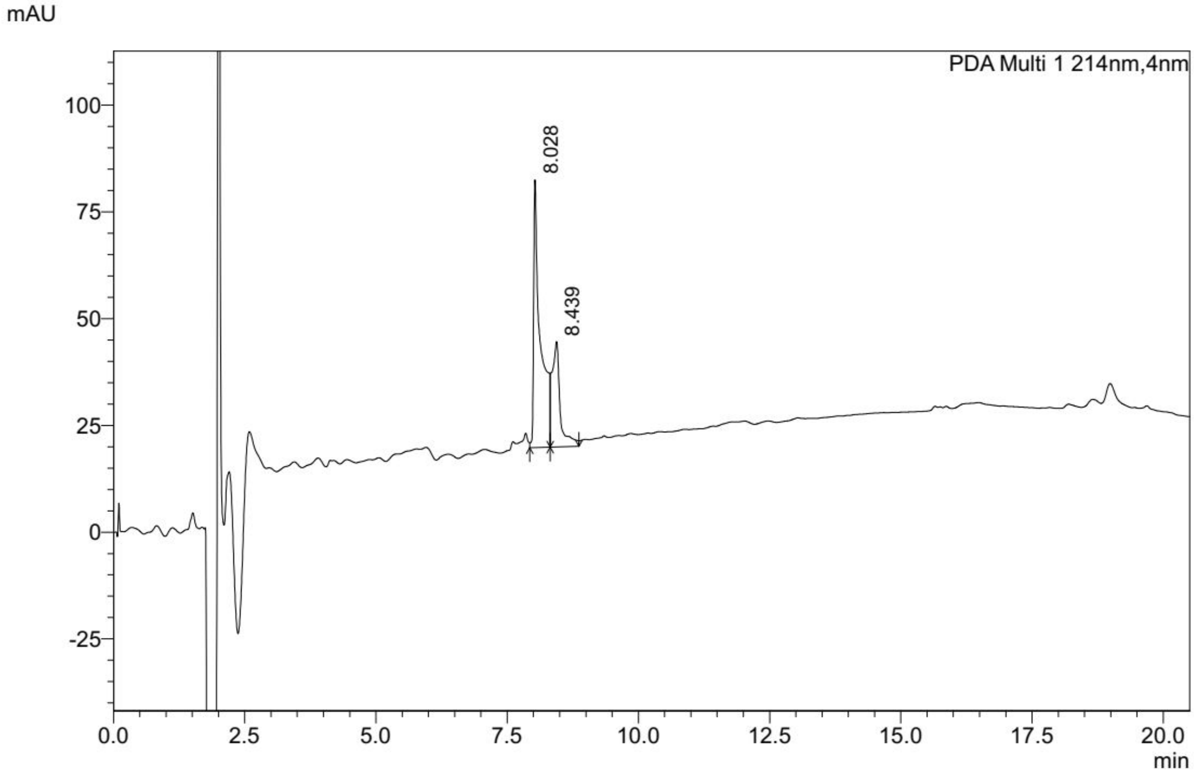

Figure S6. HPLC chromatogram of inhibitor 1, temperature of analysis 30 °C.

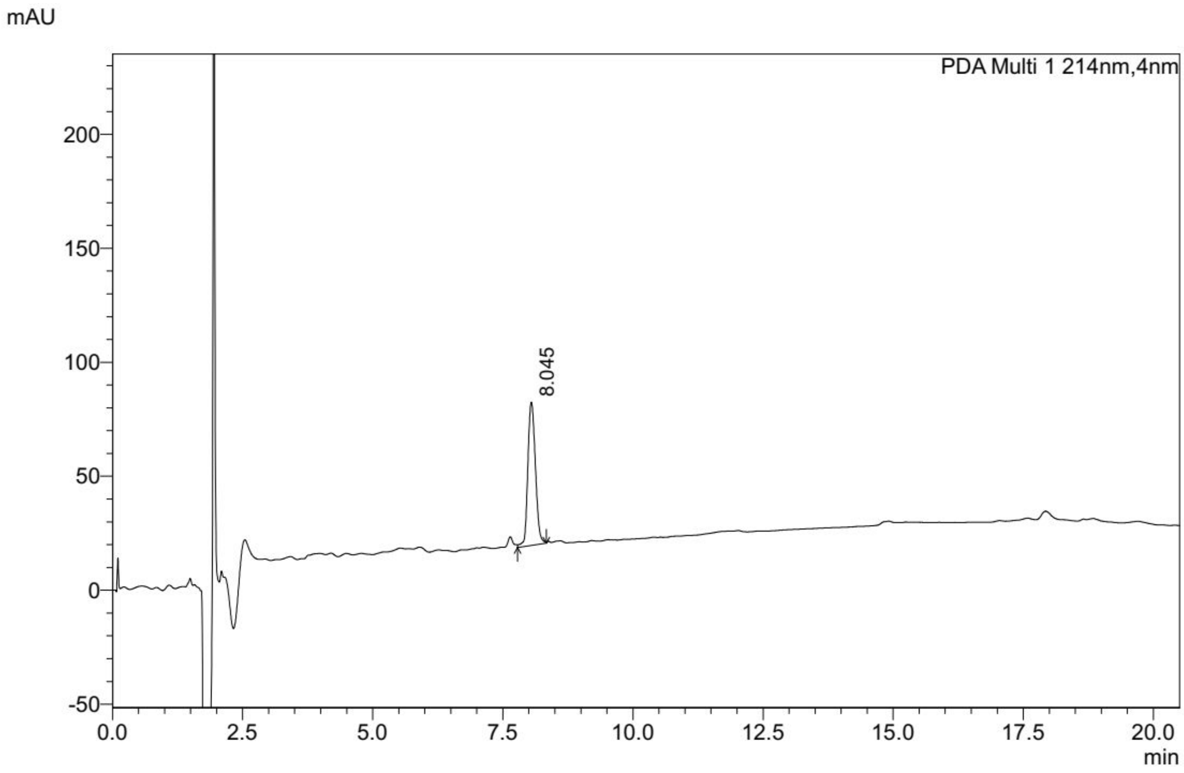

Figure S7. HPLC chromatogram of inhibitor 1, temperature of analysis 50 °C.

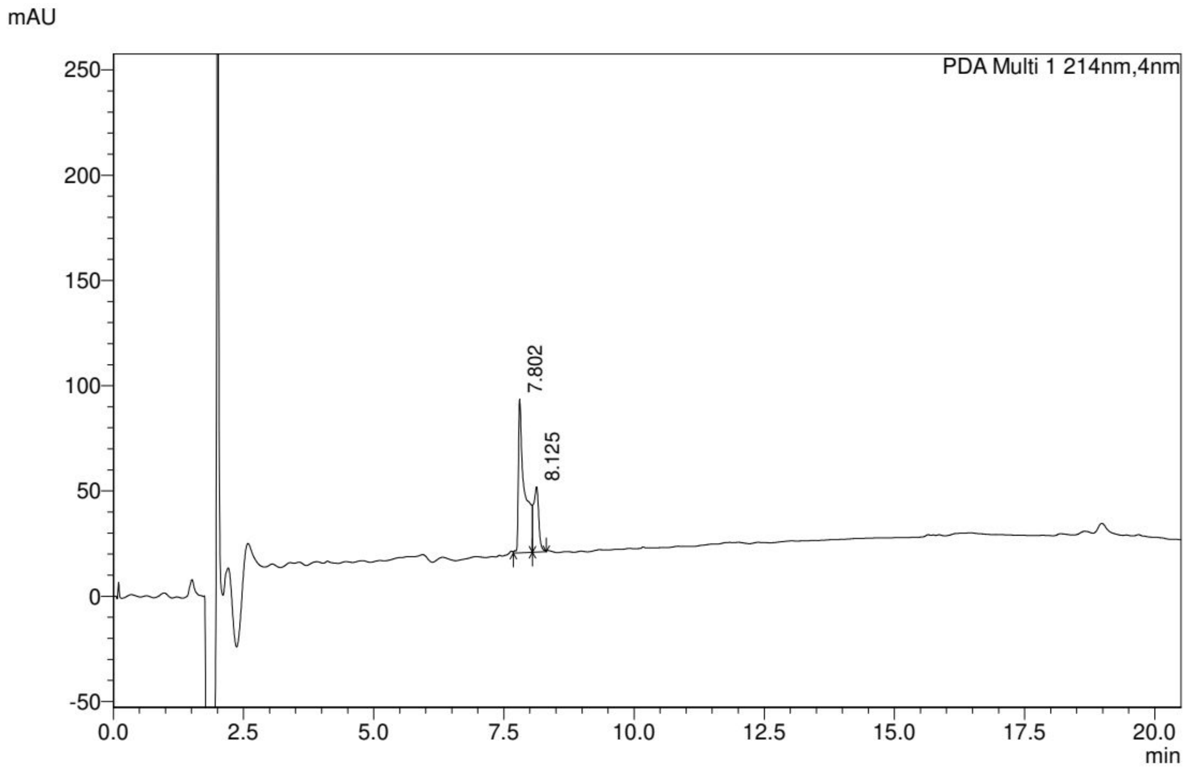

Figure S8. HPLC chromatogram of inhibitor 2, temperature of analysis 30 °C.

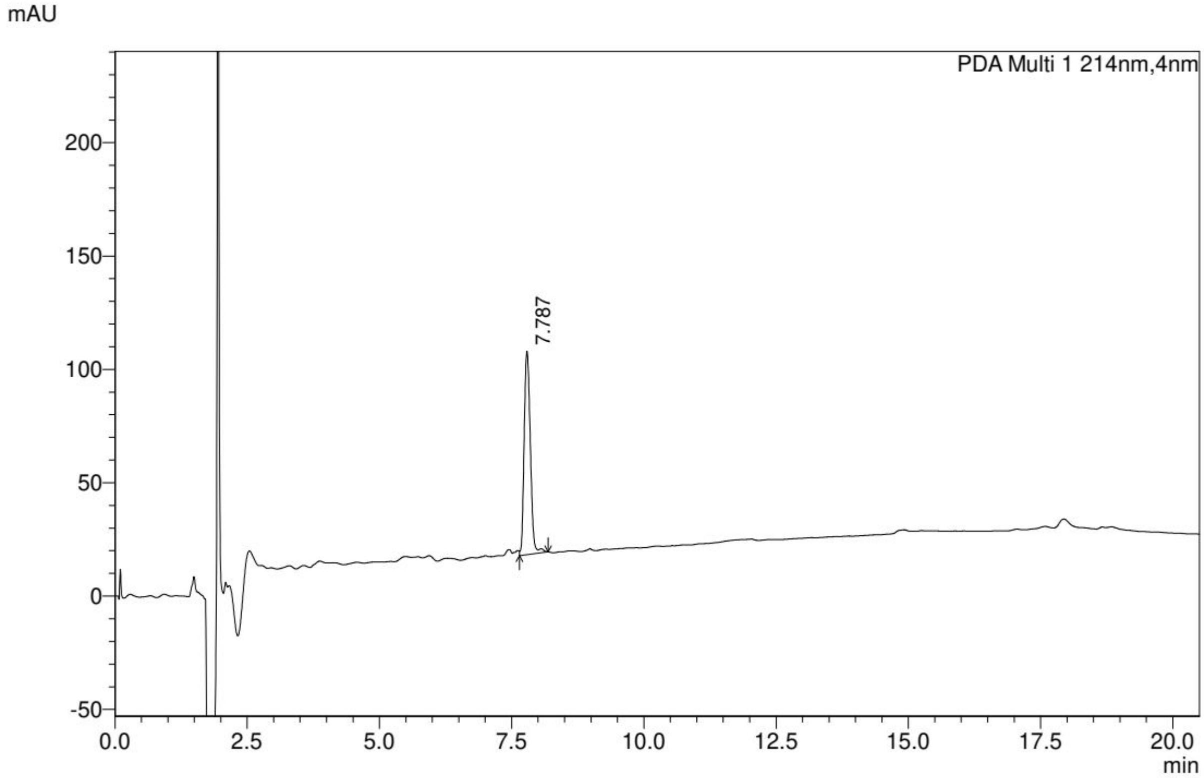

Figure S9. HPLC chromatogram of inhibitor 2, temperature of analysis 50 °C.

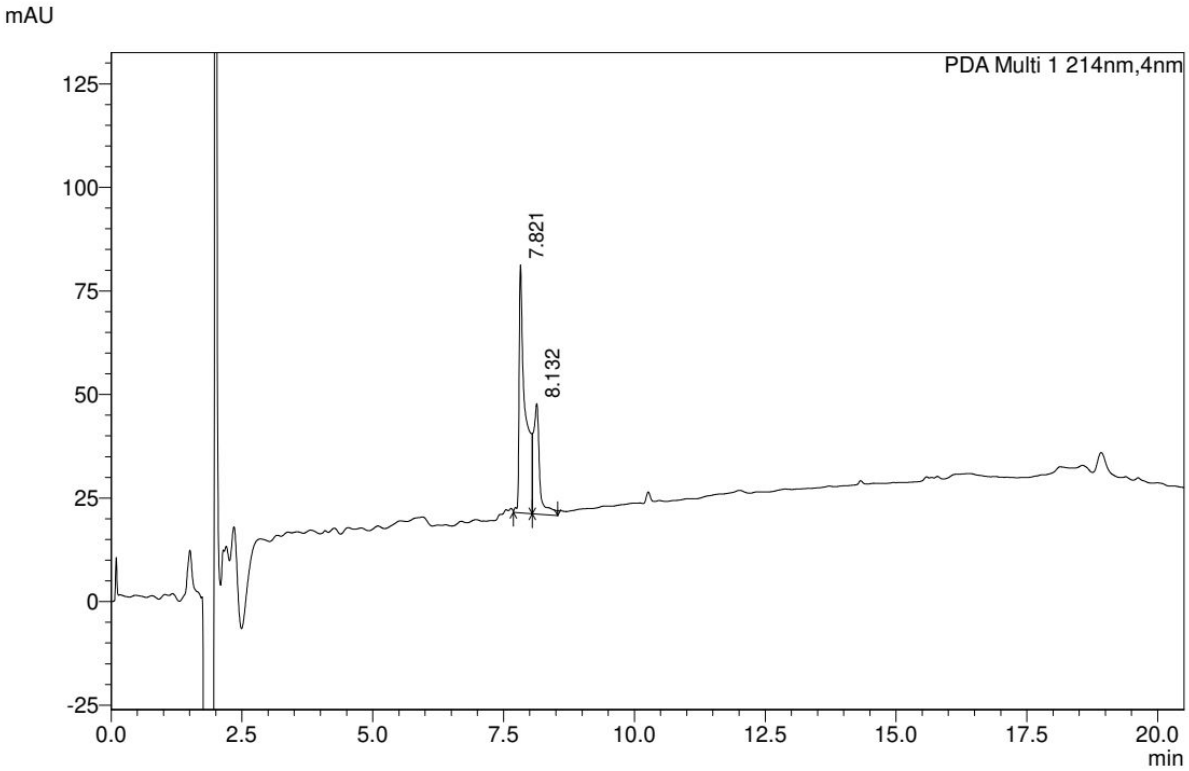

Figure S10. HPLC chromatogram of inhibitor 3, temperature of analysis 30 °C.

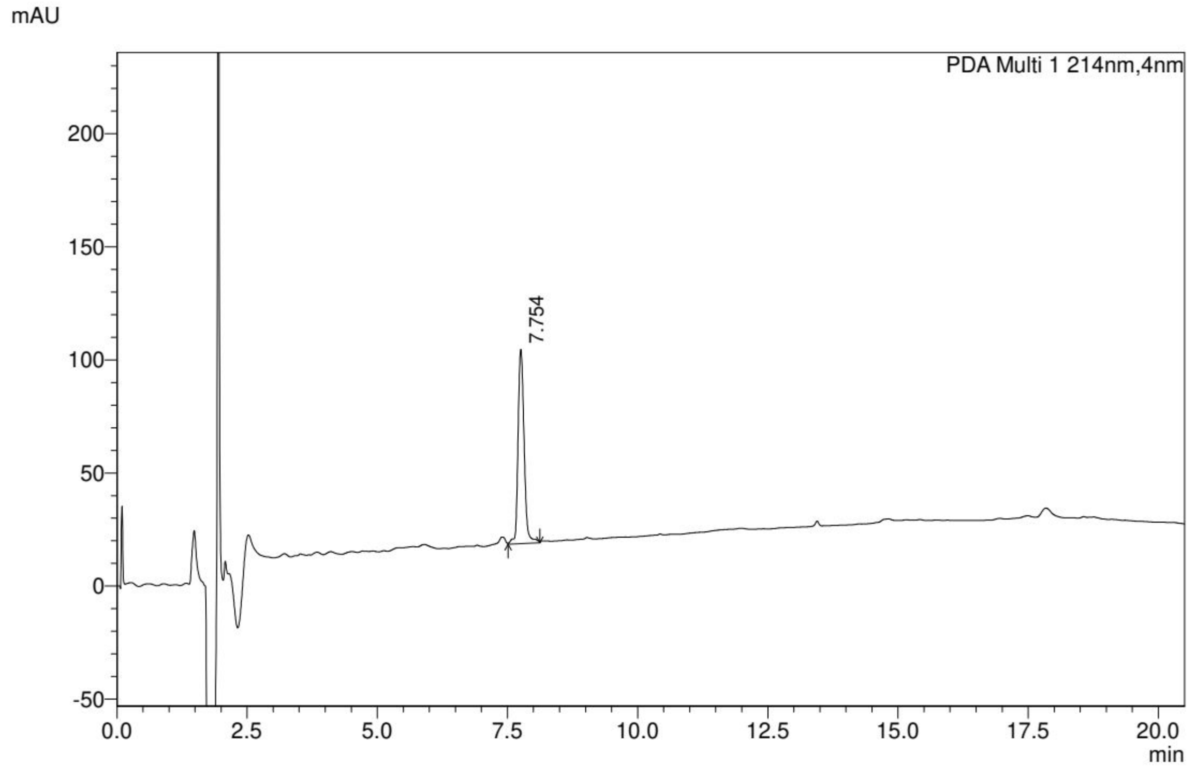

Figure S11. HPLC chromatogram of inhibitor 3, temperature of analysis 50 °C.

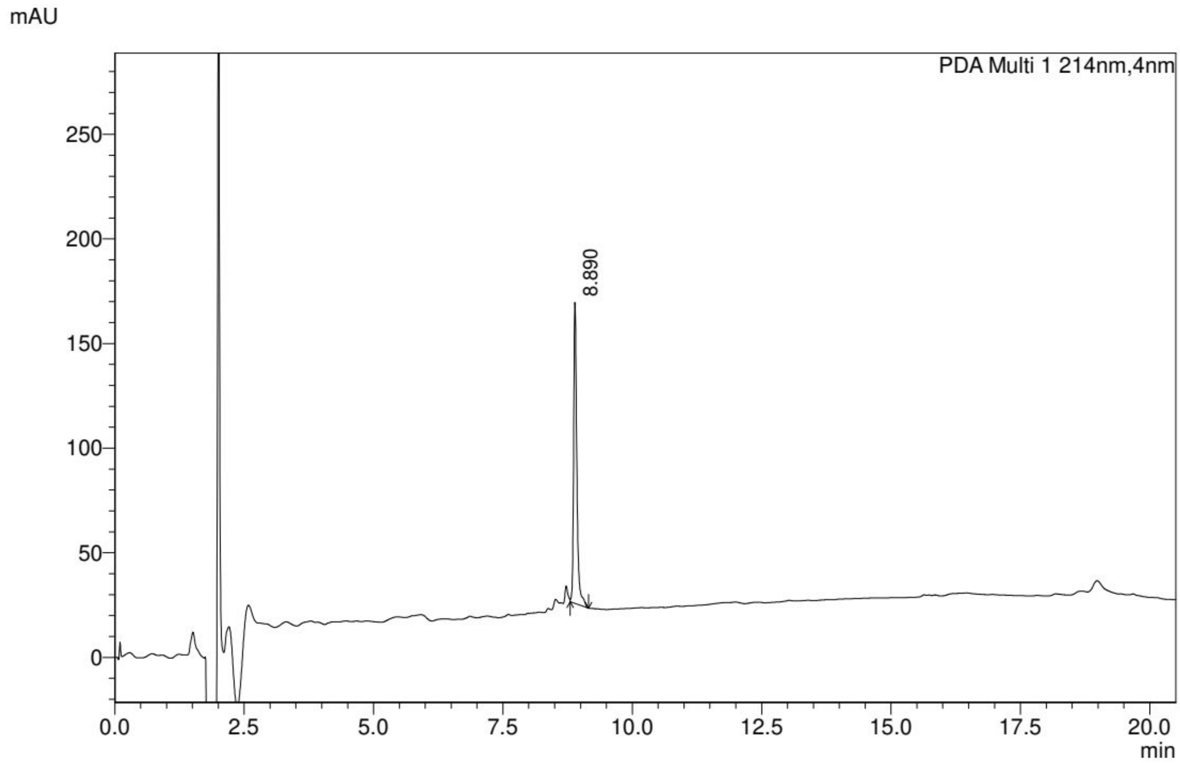

Figure S12. HPLC chromatogram of inhibitor 4, temperature of analysis 30 °C.

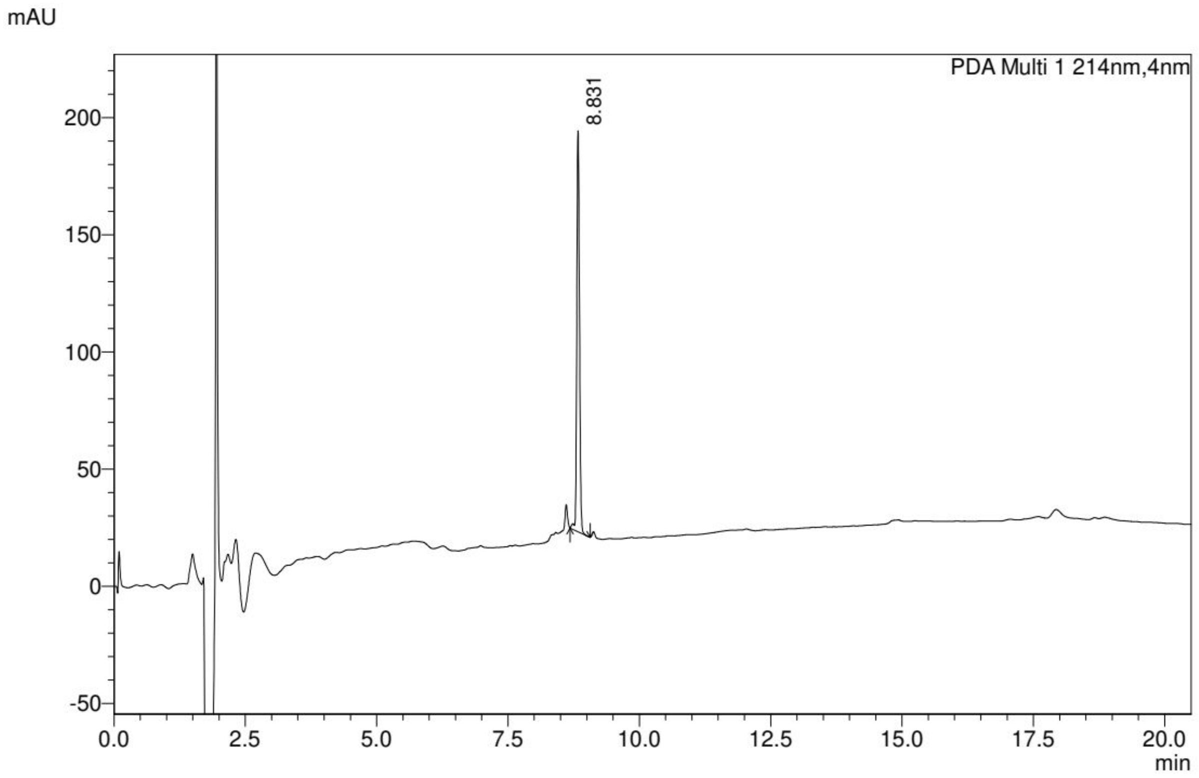

Figure S13. HPLC chromatogram of inhibitor 4, temperature of analysis 50 °C.

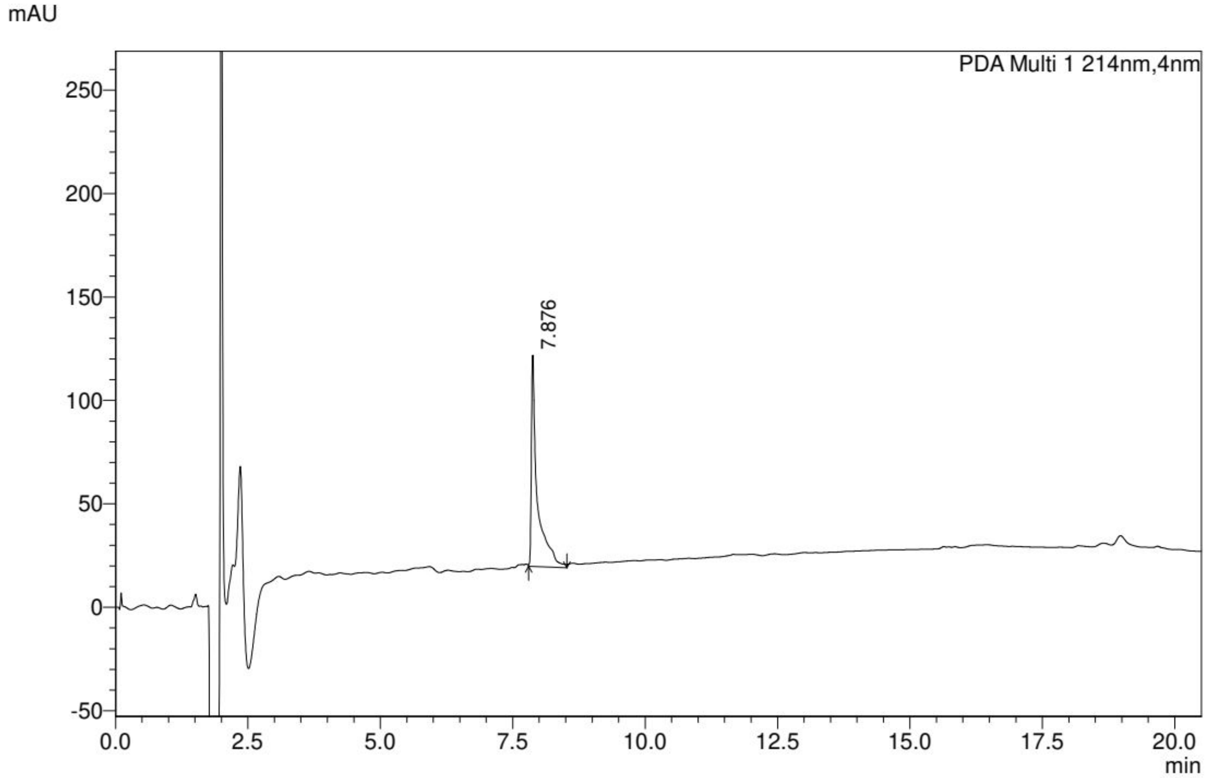

Figure S14. HPLC chromatogram of inhibitor 5, temperature of analysis 30 °C.

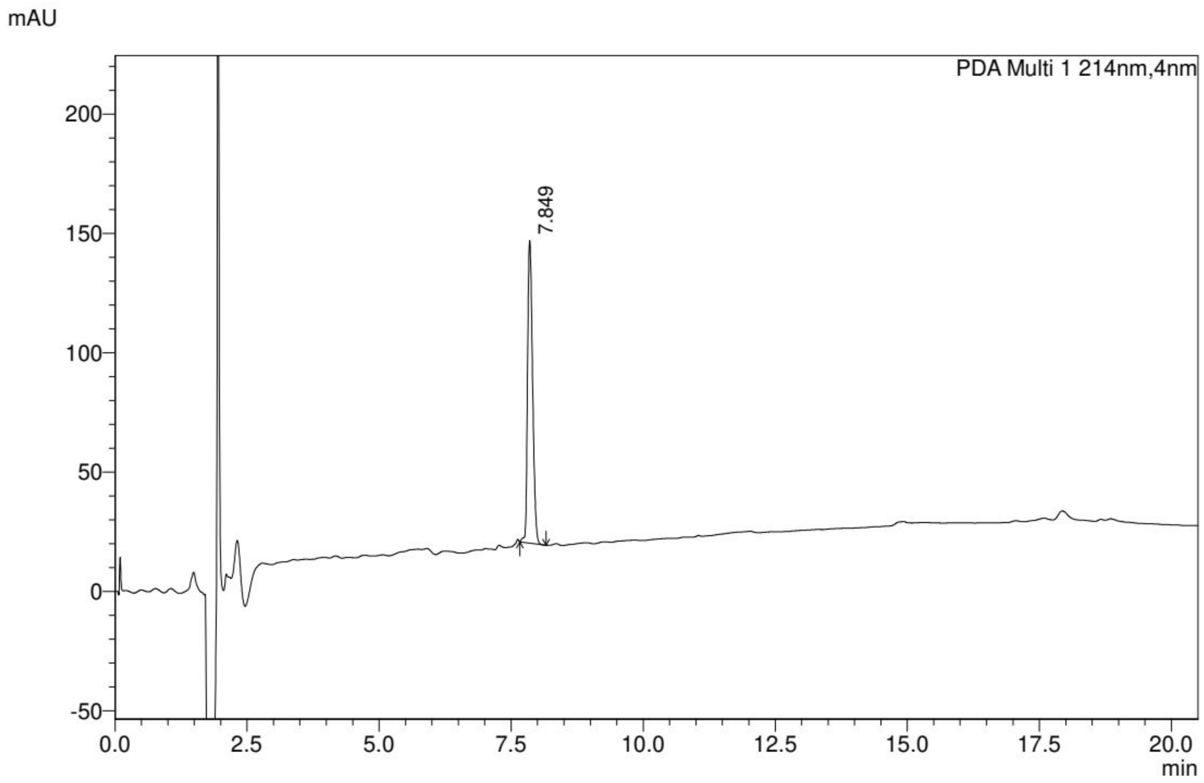

Figure S15.HPLC chromatogram of inhibitor 5, temperature of analysis 50 °C.

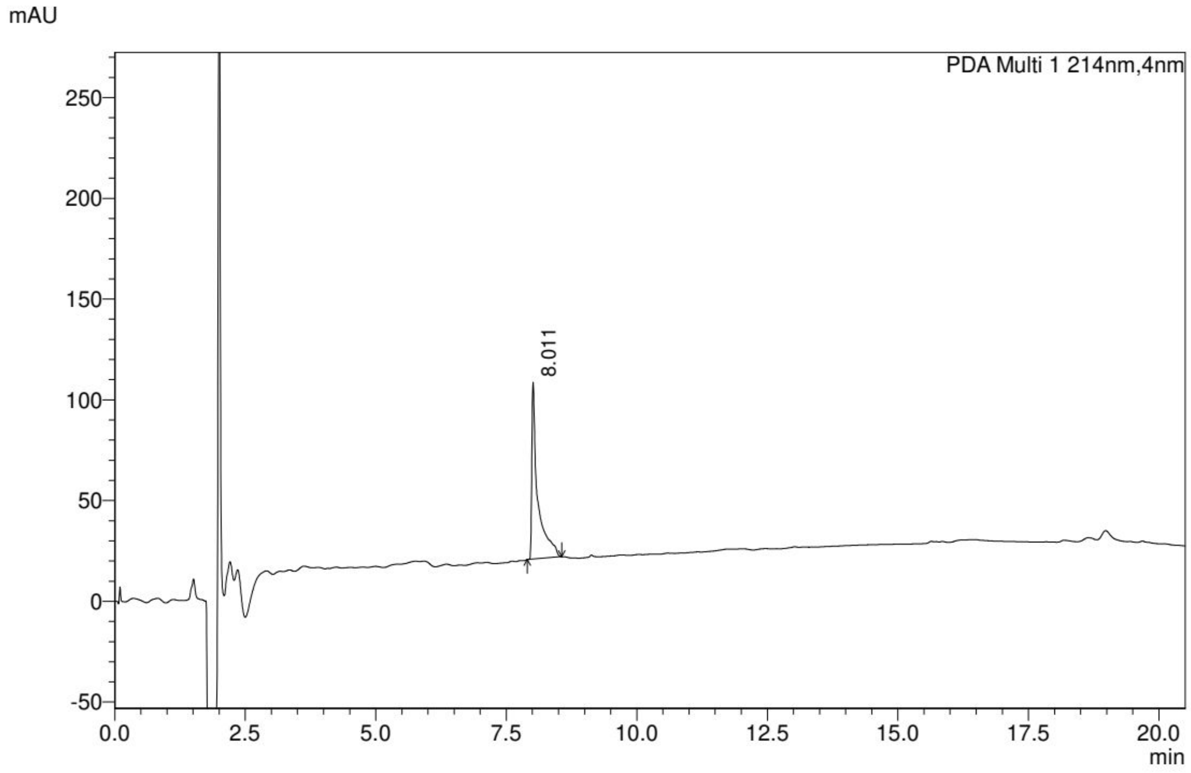

Figure S16. HPLC chromatogram of inhibitor 6, temperature of analysis 30 °C.

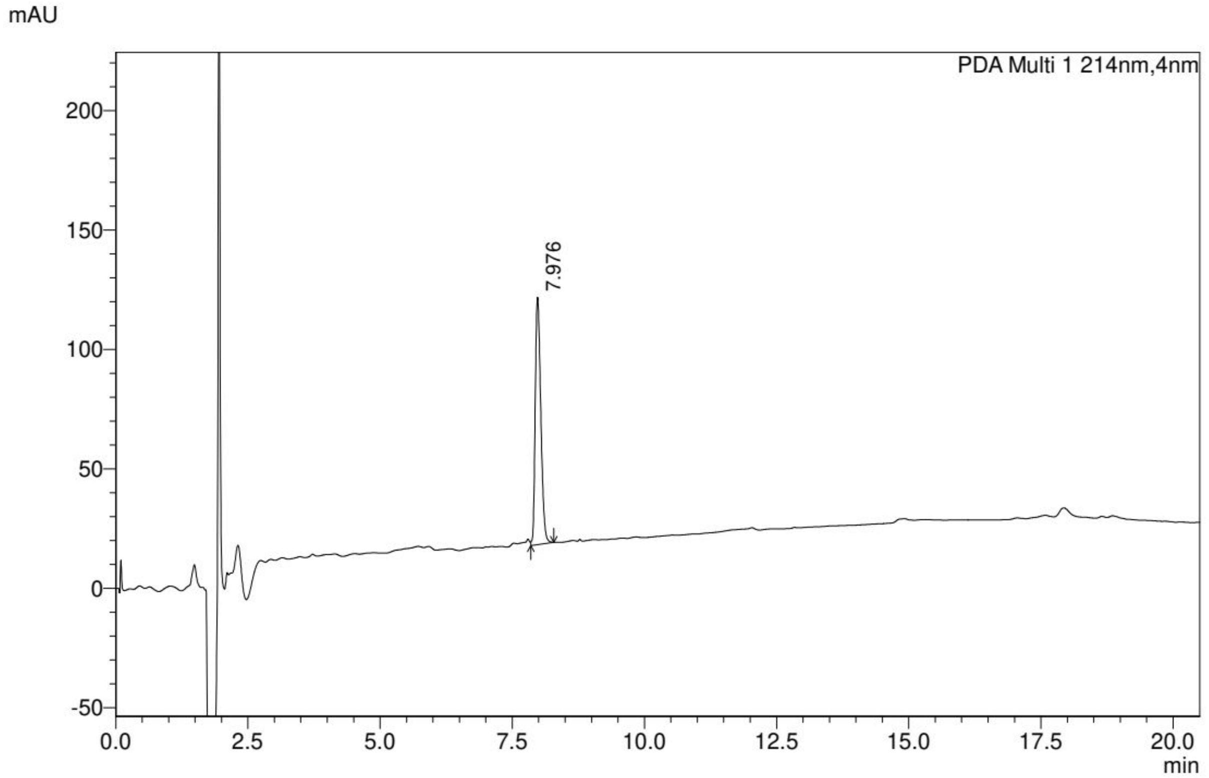

Figure S17. HPLC chromatogram of inhibitor 6, temperature of analysis 50 °C.

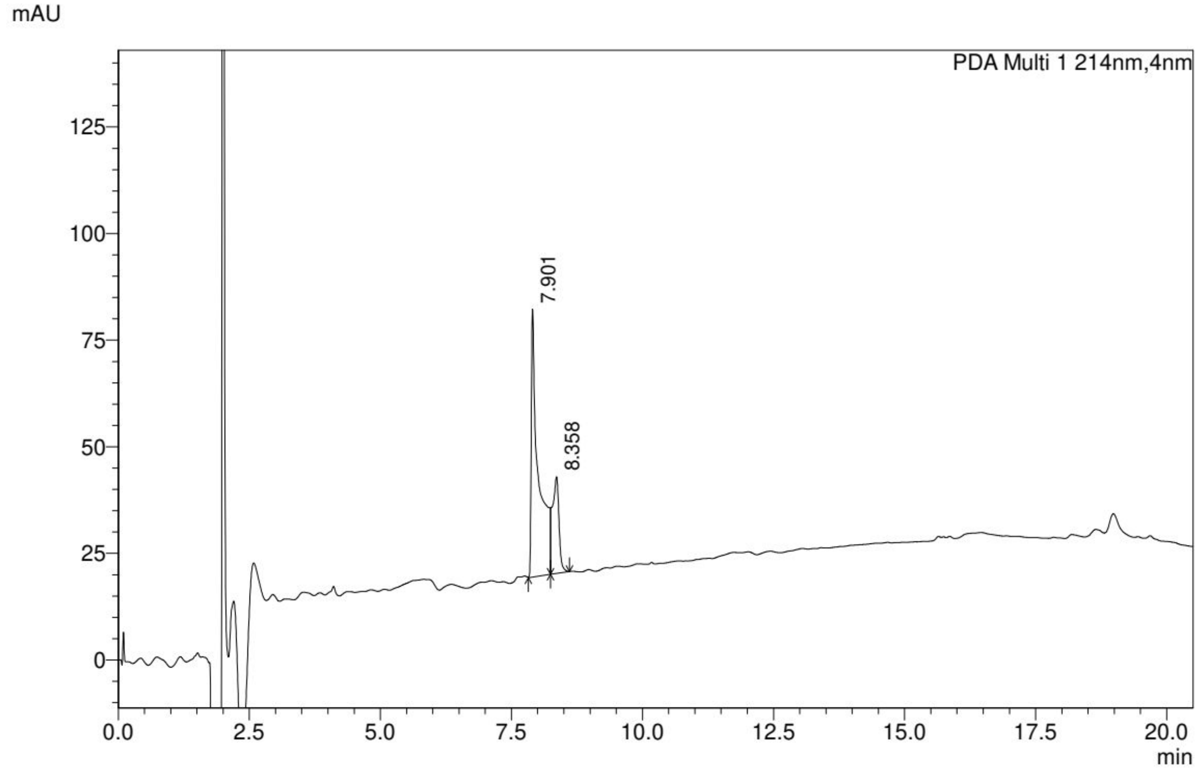

Figure S18. HPLC chromatogram of Fittler inhibitor (FI), temperature of analysis 30 °C.

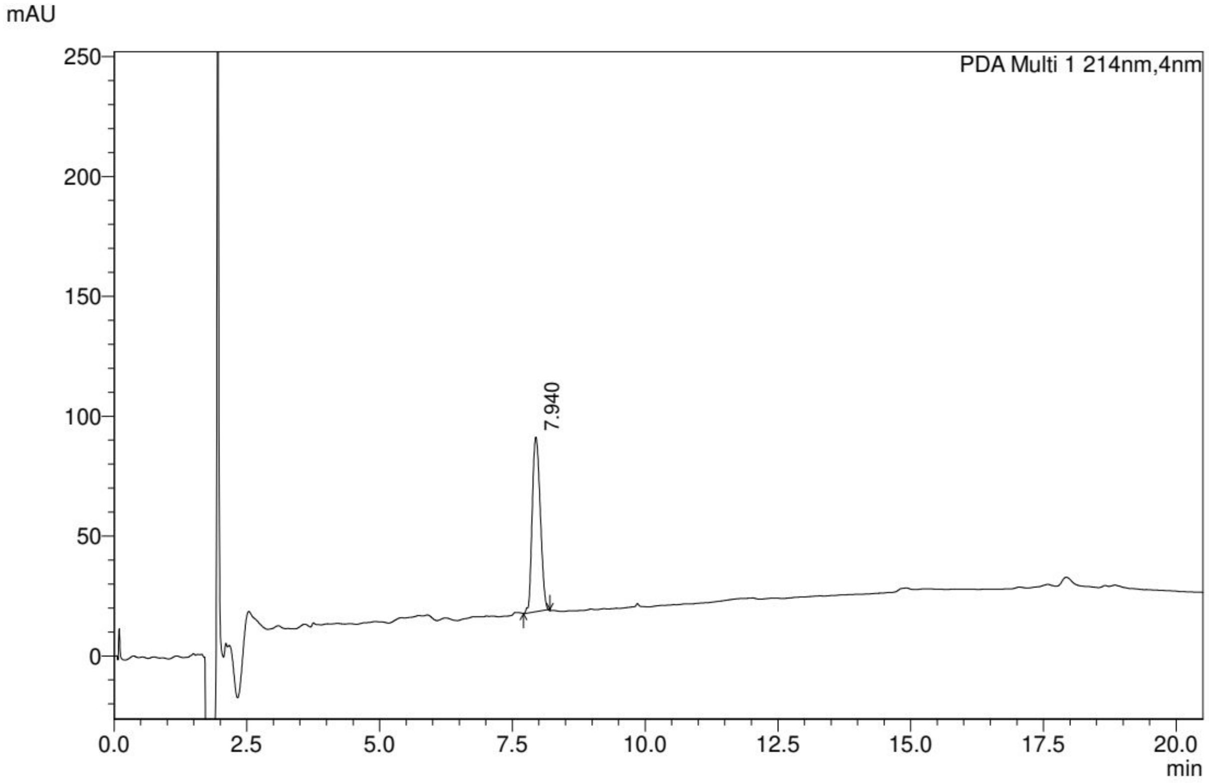

Figure S19. HPLC chromatogram of Fittler inhibitor (FI), temperature of analysis 50 °C.

MS analyses of synthesized peptides

MALDI MS (Autoflex maX MALDI-TOF spectrometer, Bruker Daltonics, Germany) using an α-cyano-4-hydroxycinnamic acid and/or 2,5-dihydroxybenzoic acid matrix

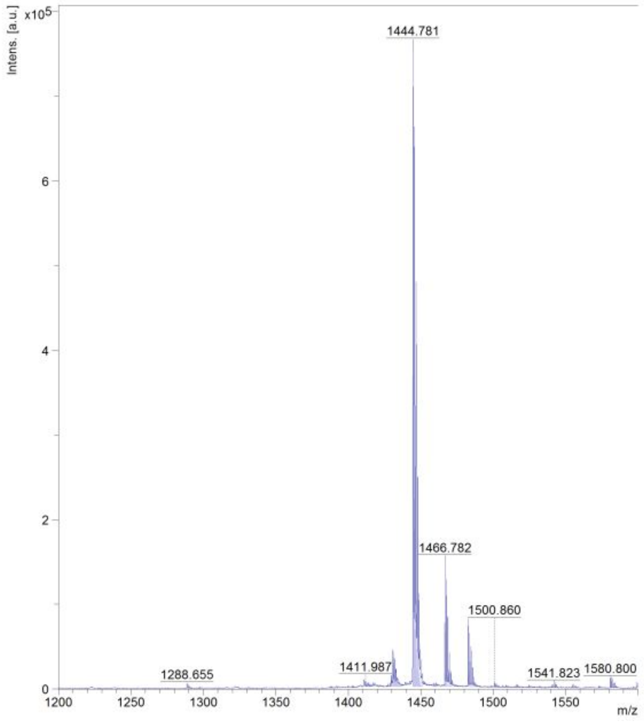

Figure S20. MS analysis of peptide 1. DHB matrix.

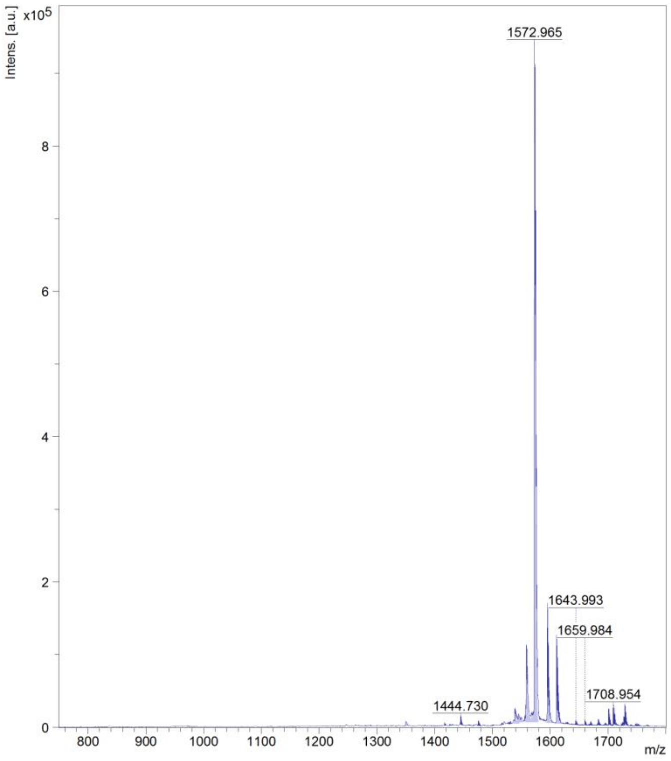

Figure S21. MS analysis of peptide 2. DHB matrix.

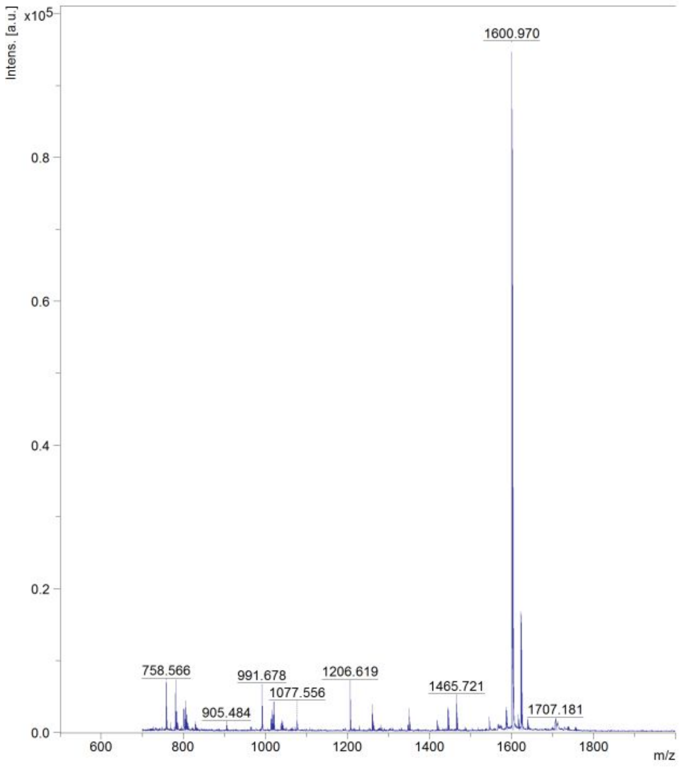

Figure S22. MS analysis of peptide 3. CCA matrix.

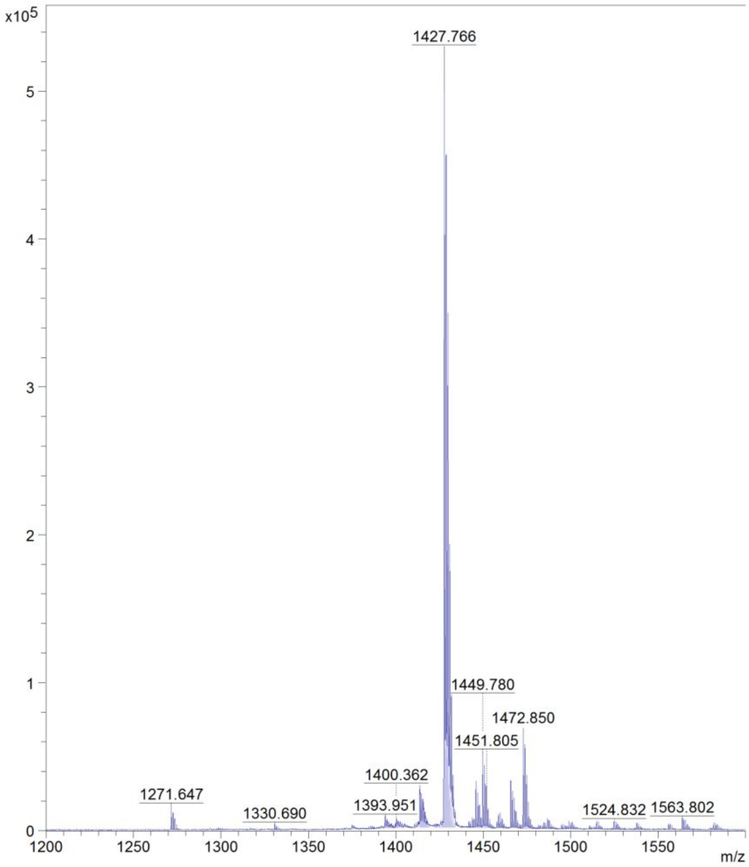

Figure S23. MS analysis of peptide 4. CCA matrix.

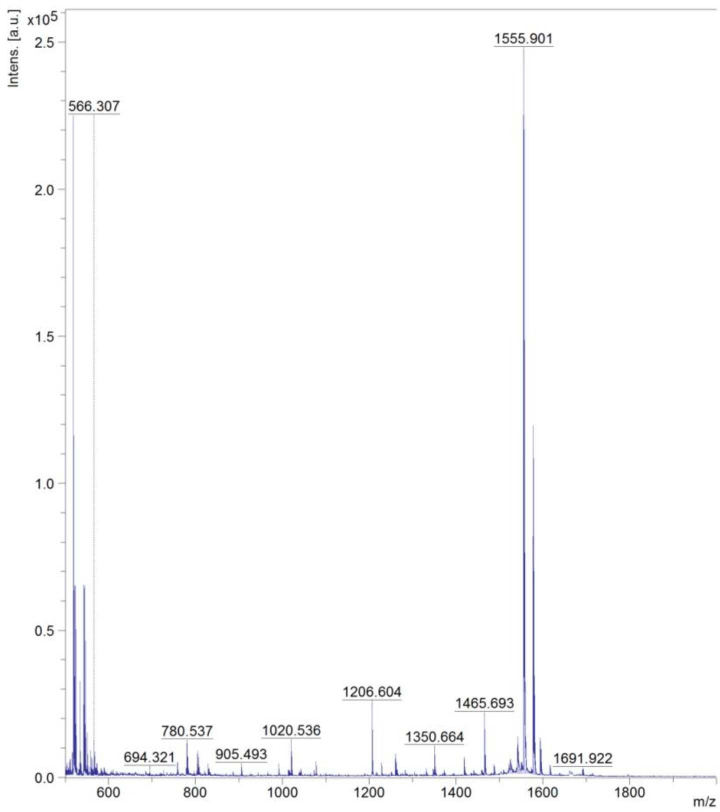

Figure S24. MS analysis of peptide 5. DHB matrix.

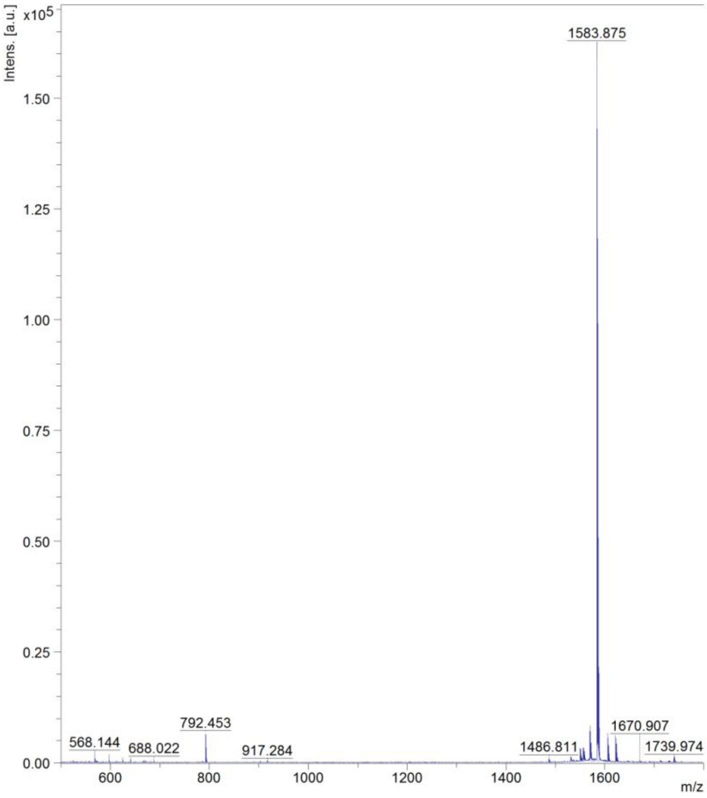

Figure S25. MS analysis of peptide 6. CCA matrix.

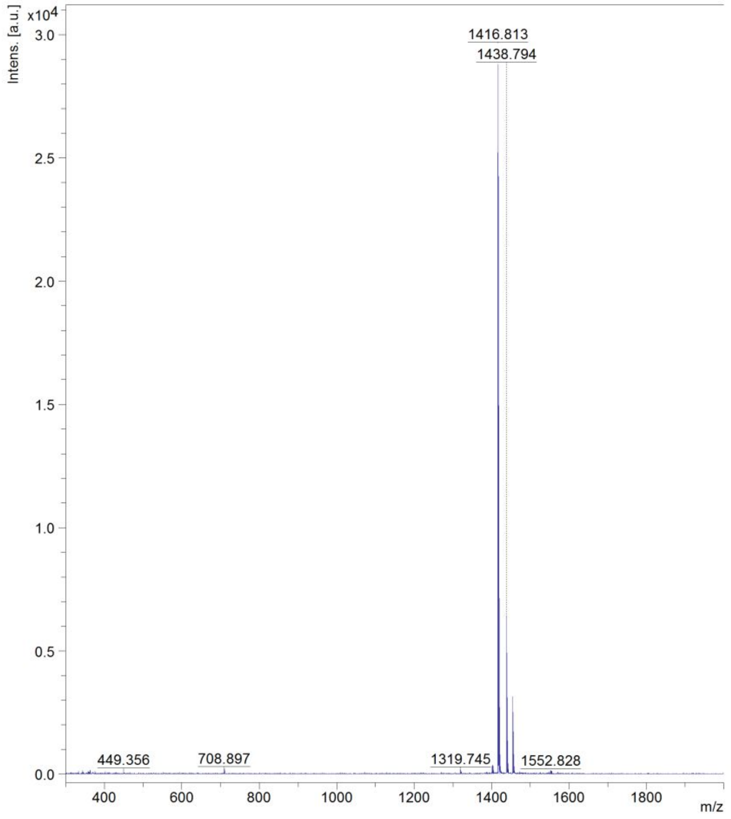

Figure S26. MS analysis of Fittler inhibitor (FI). DHB matrix.

N-Fmoc-4-(tert-butoxycarbonyl)amino-L-phenylalanine MS and HPL analyses

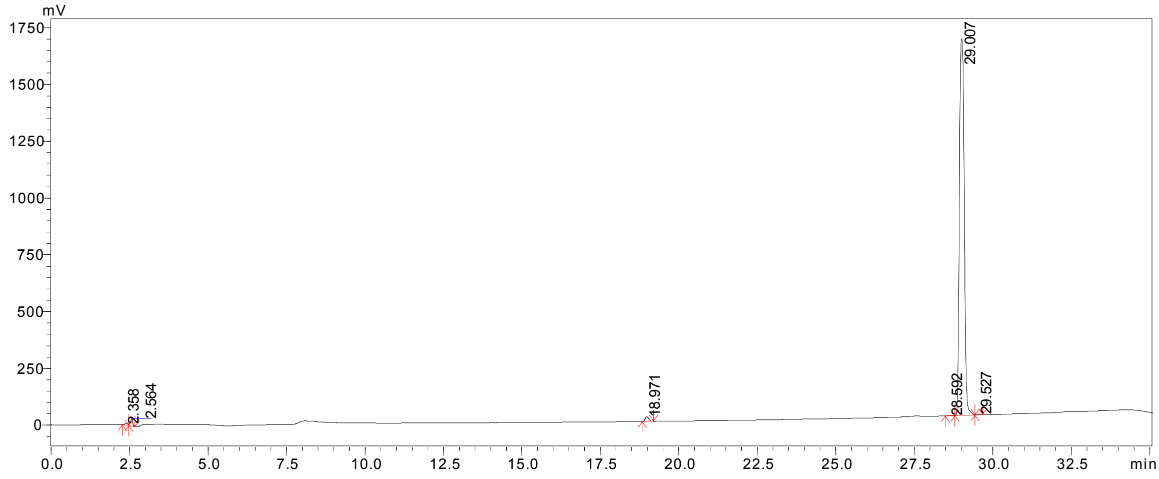

Figure S27. HPLC chromatograms obtained for N-Fmoc-4-(tert-butoxycarbonyl)amino-L-phenylalanine (Fmoc-Phe(4-NH-Boc)). HPLC conditions: Shimadzu Prominence UFLC with a Kinetex XB-C18 column 150 × 4.6 mm, 5 µm, linear gradient 10%–90% B for 30 min, 1 ml min−1, 226 nm

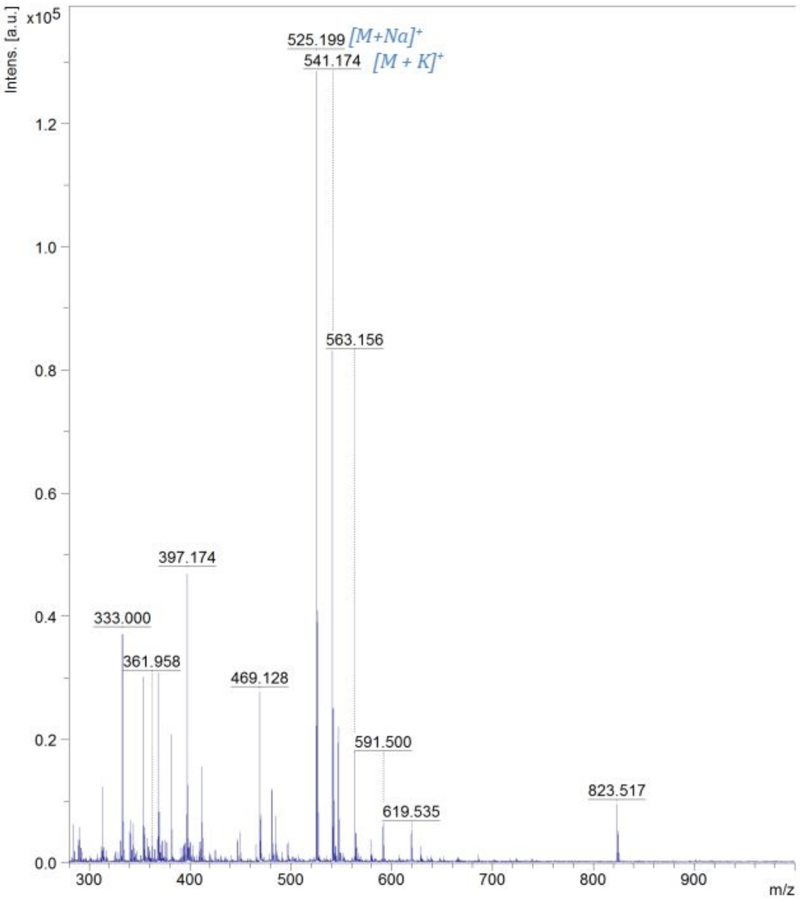

Figure S28. MS analysis of Fmoc-Phe(4-NH-Boc)

Serum stability HPLC chromatograms

3

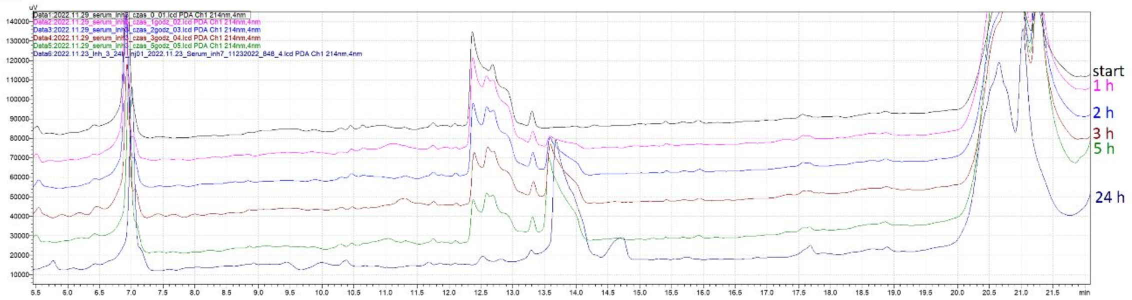

5

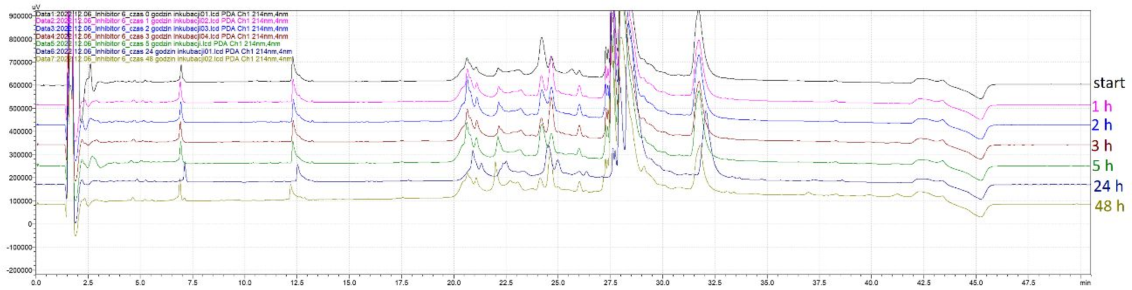

6

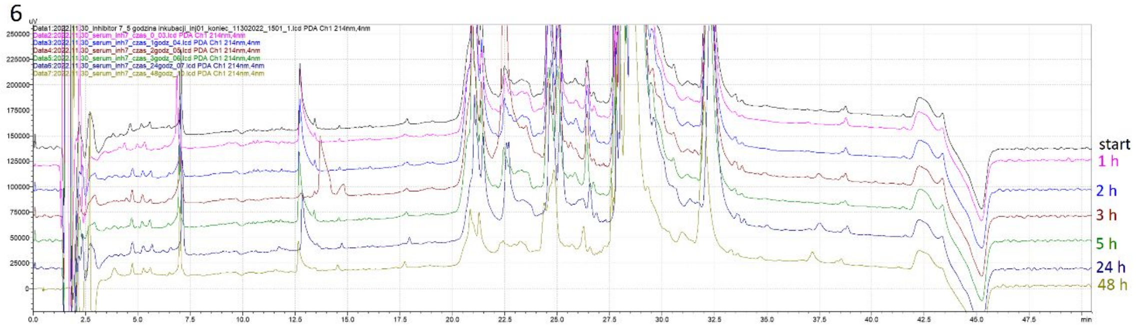

FI

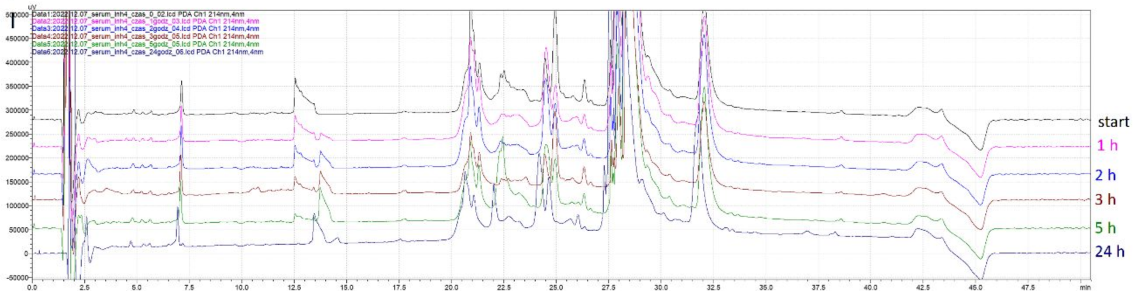

Figure S29. HPLC chromatograms obtained for inhibitor 3, 5, 6 and FI before and after incubation with human serum for 1 h, 2h, 3 h, 5h, 24 h (3 and FI) and 48h (5 and 6). Shimadzu Prominence-I RP-HPLC (linear gradient 10-90% phase B, 40 min., Kinetex 5 µm XB-C18 100Å 150 x 4.6 mm) 214nm.

References

(1) Furka, A.; Sebestyen, F. .; Asgedom, M. .; Dibo, G. General Method for Rapid Synthesis of Multicomponent Peptide Mixtures. *Int. J. Pept. Protein Res.* **1991**, *37* (6), 487–493. https://doi.org/10.1111/j.1399-3011.1991.tb00765.x.

(2) Gitlin-Domagalska, A.; Dębowski, D.; Łęgowska, A.; Stirnberg, M.; Okońska, J.; Gütschow, M.; Rolka, K. Design and Chemical Syntheses of Potent Matriptase-2 Inhibitors Based on Trypsin Inhibitor SFTI-1 Isolated from Sunflower Seeds. *Biopolymers* **2017**, *108* (6), 1–11. https://doi.org/10.1002/bip.23031.

(3) Ozturk, S.; Forneris, C. C.; Nguy, A. K. L.; Sorensen, E. J.; Seyedsayamdost, M. R. Modulating OxyB-Catalyzed Cross-Coupling Reactions in Vancomycin Biosynthesis by Incorporation of Diverse d -Tyr Analogues. *J. Org. Chem.* **2018**, *83* (13), 7309–7317. https://doi.org/10.1021/acs.joc.8b00916.

(4) Lewandowska-Goch, M. A.; Kwiatkowska, A.; Łepek, T.; Ly, K.; Navals, P.; Gagnon, H.; Dory, Y. L.; Prahl, A.; Day, R. Design and Structure-Activity Relationship of a Potent Furin Inhibitor Derived from Influenza Hemagglutinin. *ACS Med. Chem. Lett.* **2021**, *12* (3), 365–372. https://doi.org/10.1021/acsmedchemlett.0c00386.

(5) Brooks, H. B.; Geeganage, S.; Kahl, S. D.; Montrose, C.; Sittampalam, S.; Smith, M. C.; Weidner, J. R. Basics of Enzymatic Assays for HTS. *Assay Guid. Man.* **2004**, No. Md, 1–12.

(6) de Veer, S. J.; White, A. M.; Craik, D. J. Sunflower Trypsin Inhibitor-1 (SFTI-1): Sowing Seeds in the Fields of Chemistry and Biology. *Angew. Chemie - Int. Ed.* **2021**, *60* (15), 8050–8071. https://doi.org/10.1002/anie.202006919.

(7) Bourne, G. L.; Grainger, D. J. Development and Characterisation of an Assay for Furin Activity. *J. Immunol. Methods* **2011**, *364* (1–2), 101–108. https://doi.org/10.1016/j.jim.2010.11.008.

(8) Łęgowska, A.; Dębowski, D.; Łukajtis, R.; Wysocka, M.; Czaplewski, C.; Lesner, A.; Rolka, K. Implication of the Disulfide Bridge in Trypsin Inhibitor SFTI-1 in Its Interaction with Serine Proteinases. *Bioorganic Med. Chem.* **2010**, *18* (23), 8188–8193. https://doi.org/10.1016/j.bmc.2010.10.014.

(9) D.A. Case, D.S. Cerutti, T.E. Cheatham, III, T.A. Darden, R.E. Duke, T.J. Giese, H. Gohlke, A.W. Goetz, D.; Greene, N. Homeyer, S. Izadi, A. Kovalenko, T.S. Lee, S. LeGrand, P. Li, C. Lin, J. Liu, T. Luchko, R. L.; D. Mermelstein, K.M. Merz, G. Monard, H. Nguyen, I. Omelyan, A. Onufriev, F. Pan, R. Qi, D.R. Roe, A.; Roitberg, C. Sagui, C.L. Simmerling, W.M. Botello-Smith, J. Swails, R.C. Walker, J. Wang, R.M. Wolf, X.; Wu, L. Xiao, D. M. Y. and P. A. K. AMBER 2017. University of California, San Francisco 2017.

(10) Nguyen, H.; Maier, J.; Huang, H.; Perrone, V.; Simmerling, C. Folding Simulations for Proteins with Diverse Topologies Are Accessible in Days with a Physics-Based Force Field and Implicit Solvent. *J. Am. Chem. Soc.* **2014**, *136* (40), 13959–13962. https://doi.org/10.1021/ja5032776.

(11) Jorgensen, W. L.; Chandrasekhar, J.; Madura, J. D.; Impey, R. W.; Klein, M. L. Comparison of Simple Potential Functions for Simulating Liquid Water. *J. Chem. Phys.* **1983**, *79* (2), 926–935. https://doi.org/10.1063/1.445869.

(12) Ryckaert, J. P.; Ciccotti, G.; Berendsen, H. J. C. Numerical Integration of the Cartesian Equations of Motion of a System with Constraints: Molecular Dynamics of n-Alkanes. *J. Comput. Phys.* **1977**, *23* (3), 327–341. https://doi.org/10.1016/0021-9991(77)90098-5.

(13) Essmann, U.; Perera, L.; Berkowitz, M. L.; Darden, T.; Lee, H.; Pedersen, L. G. A Smooth Particle Mesh Ewald Method. *J. Chem. Phys.* **1995**, *103* (19), 8577–8593.

https://doi.org/10.1063/1.470117.

(14) Homeyer, N.; Gohlke, H. Free Energy Calculations by the Molecular Mechanics Poisson-Boltzmann Surface Area Method. *Mol. Inform.* **2012**, *31* (2), 114–122. https://doi.org/10.1002/minf.201100135.

(15) Roe, D. R.; Cheatham, T. E. PTRAJ and CPPTRAJ: Software for Processing and Analysis of Molecular Dynamics Trajectory Data. *J. Chem. Theory Comput.* **2013**, *9* (7), 3084–3095. https://doi.org/10.1021/ct400341p.

(16) Humphrey, W.; Dalke, A.; Schulten, K. VMD: Visual Molecular Dynamics. *J. Mol. Graph.* **1996**, *14* (October 1995), 33–38. https://doi.org/10.1016/0263-7855(96)00018-5.

(17) Kálmán, A.; Thunecke, F.; Schmidt, R.; Schiller, P. W.; Horváth, C. Isolation and Identification of Peptide Conformers by Reversed-Phase High-Performance Liquid Chromatography and NMR at Low Temperature. *J. Chromatogr. A* **1996**, *729* (1–2), 155–171. https://doi.org/10.1016/0021-9673(95)01059-9.

(18) Winter, D.; Pipkorn, R.; Lehmann, W. D. Separation of Peptide Isomers and Conformers by Ultra Performance Liquid Chromatography. *J. Sep. Sci.* **2009**, *32* (8), 1111–1119. https://doi.org/10.1002/jssc.200800691.
